# SNF-NN: computational method to predict drug-disease interactions using similarity network fusion and neural networks

**DOI:** 10.1186/s12859-020-03950-3

**Published:** 2021-01-22

**Authors:** Tamer N. Jarada, Jon G. Rokne, Reda Alhajj

**Affiliations:** 1 Department of Computer Science, University of Calgary, Calgary, AB Canada; 2 Department of Computer Engineering, Istanbul Medipol University, Istanbul, Turkey; 3 Department of Health Informatics, University of Southern Denmark, Odense, Denmark

**Keywords:** Computational drug repositioning, Drug similarity measures, Similarity network fusion, Machine learning, Deep learning

## Abstract

**Background:**

Drug repositioning is an emerging approach in pharmaceutical research for identifying novel therapeutic potentials for approved drugs and discover therapies for untreated diseases. Due to its time and cost efficiency, drug repositioning plays an instrumental role in optimizing the drug development process compared to the traditional de novo drug discovery process. Advances in the genomics, together with the enormous growth of large-scale publicly available data and the availability of high-performance computing capabilities, have further motivated the development of computational drug repositioning approaches. More recently, the rise of machine learning techniques, together with the availability of powerful computers, has made the area of computational drug repositioning an area of intense activities.

**Results:**

In this study, a novel framework SNF-NN based on deep learning is presented, where novel drug-disease interactions are predicted using drug-related similarity information, disease-related similarity information, and known drug-disease interactions. Heterogeneous similarity information related to drugs and disease is fed to the proposed framework in order to predict novel drug-disease interactions. SNF-NN uses similarity selection, similarity network fusion, and a highly tuned novel neural network model to predict new drug-disease interactions. The robustness of SNF-NN is evaluated by comparing its performance with nine baseline machine learning methods. The proposed framework outperforms all baseline methods ($AUC-ROC$ = 0.867, and $AUC-PR$=0.876) using stratified 10-fold cross-validation. To further demonstrate the reliability and robustness of SNF-NN, two datasets are used to fairly validate the proposed framework’s performance against seven recent state-of-the-art methods for drug-disease interaction prediction. SNF-NN achieves remarkable performance in stratified 10-fold cross-validation with $AUC-ROC$ ranging from 0.879 to 0.931 and $AUC-PR$ from 0.856 to 0.903. Moreover, the efficiency of SNF-NN is verified by validating predicted unknown drug-disease interactions against clinical trials and published studies.

**Conclusion:**

In conclusion, computational drug repositioning research can significantly benefit from integrating similarity measures in heterogeneous networks and deep learning models for predicting novel drug-disease interactions. The data and implementation of SNF-NN are available at http://pages.cpsc.ucalgary.ca/ tnjarada/snf-nn.php.

## Background

Humankind has always been faced with diseases of various kinds. In the past, these diseases either resulted in death, disability, or recovery, and there were no tools that would affect the outcome. A major step forward was the discovery that certain substances or drugs could alleviate the symptoms of a disease or even aid the recovery from the disease. Over time, a slate of drugs has been identified or developed for many diseases. There remains, however, a number of diseases for which no drugs have been found or developed so far.

One of the reasons is that new drug development is an expensive, lengthy, complex, and risky process. According to a very recent study, the average estimated research and development cost for new FDA-approved drugs targeting different therapeutic areas range between 0.8 and 4.5 billion USD [1]. Such a high cost is due to the expenditures associated with the high failure rate of pre-clinical trails. Moreover, the traditional *de novo* strategy to develop a new therapeutic agent includes a number of steps, which could take up to 17 years with less than 10% success rate [2].

There is a great need for developing new drugs for diseases whose current drugs have unpleasant side effects, emerging diseases such as *COVID-19*, and rare disease where a recent study reported that there are about seven thousand rare diseases without any therapeutics that affect more than 400 million people around the globe [3].

While the pharmaceutical industry has invested heavily in the research and development of new drugs, the number of newly approved drugs is also decreasing significantly because of the associated risk factors (e.g., side effects) [4]. There is, therefore, a need for innovative drug development strategies that overcome the limitations of the traditional *de novo* strategy.

In recent years, drug repositioning has emerged as a coming-on-strong alternative to the *de novo* strategy, and about 30% of new FDA-approved therapeutic agents and vaccines are due to successful drug repositioning studies [5]. Drug repositioning is the process of identifying novel therapeutic potentials for existing drugs and discovering therapies for untreated diseases. Drug repositioning strategies have played an instrumental role in optimizing the pre-clinical process of developing new drugs as well as giving a second chance to shelved, withdrawn, and failed drugs due to their time and cost efficiency.

Traditional drug repositioning studies usually focus on uncovering drug mode of action (MoA) similarities [6], revealing novel drug indications [7], investigating common characteristics between drug compounds [8], or discovering relationships between drugs and diseases [9]. A major challenge in these studies is to distinguish the actual molecular targets of a drug from hundreds to thousands of additional gene products that are affected indirectly as a result of changes in the activity of the targets. Classical statistical models and approaches are therefore ineffective for detecting the molecular targets of a drug among the massive amount of genes.

Additionally, using only one source of data is a big drawback of many traditional drug repositioning methods as these methods may only recover partial knowledge of a living organism’s behaviour. Moreover, obstacles like missing, biased, and inaccurate data may affect the performance of these models. For instance, reliable gene expression signature profiles may be hard to define for several reasons such as variations in experimental conditions (e.g., patient age, environment variables) across different experiments, which may result in a data discrepancy in gene expression signatures, contributing to having biased data. Also, there may not always be significant changes in gene expressions when these genes are used as drug targets, which can lead to having inaccurate data. Further, the lack of high-resolution structural data for drug targets makes it hard to identify potential drug-target interactions when following the chemical structure and molecule information strategy. As a result, the claims of inferences and discoveries for these methods may not be sustainable. Thus, integrating data from a variety of sources may be an approach to overcome such challenges when developing computational drug repositioning models.

Rapid advances in technology have allowed the development of effective computational models for inferring potential therapeutic applications. These computational models have been used to identify therapeutic potentials for existing drugs, which can be used to guide time-consuming and costly clinical trials. Furthermore, novel drug-disease interactions predicted by computational models can be considered for further verification (e.g., functional enrichment analysis and meta-analysis). Such models can be roughly classified into three categories [10, 11].

The first category includes data mining-based models that infer off-target drug interactions and identify novel drug repositioning candidates. Such models were built based on text mining and semantic technologies. For instance, Tari et al. [12] introduced a novel approach that uses text mining to retrieve data from publicly available sources in order to construct a set of logical facts. The logical facts were then used along with logical rules that represent drug mechanism properties to build an automated reasoning model for identifying therapeutic potentials and novel indications for existing drugs. Moreover, Rastegar-Mojarad et al. [13] developed an approach that used text-mined data to infer drug-gene and gene-disease semantic predictions. Then, the semantic predictions were used to compile a ranked list of potential drug-disease pairs. Furthermore, Zhu et al. [14] utilized Clinical Pharmacogenomics (PGx) data to build pharmacogenomics Web Ontology Language (WOL) profiles. WOL profiles were then used to identify pharmacogenomics associations for FDA approved breast cancer drugs.

The second category refers to network-based models that capture unknown drug-disease interactions relying on heterogeneous networks of biological and biomedical entities. Such models used network analysis techniques (e.g., bipartite graph, clustering, network centrality measures) to discover informative relationships. For instance, Li and Lu [15] built a supervised learning bipartite graph model to capture the implicit information between drug targets and thereby identifying drug-target indications based on drug pairwise similarity. Moreover, Tan et al. [16] constructed a drug-drug interaction network, which then was clustered into modules based on mode-of-action to identify novel drug target indications. Furthermore, Luo et al. [17] introduced a novel method named MBiRW based on comprehensive similarity measures and bi-random walk to identify potential drug repositioning applications.

The third category covers machine learning-based models that identify novel drug-disease interactions and potential therapeutic uses of existing drugs. Such models used classification techniques [e.g., Logistic Regression (LR), Naïve Bayesian (NB), Support Vector Machines (SVM), Random Forest (RF)], collaborative filtering [e.g., Matrix Factorization (MF)], and more recently deep learning [e.g., Neural Network (NN), Autoencoder (AE)] for binary classification, multiclass classification, and values prediction. For instance, Gottlieb et al. [18], Yang and Agarwal [19], Wang et al. [20], and Oh et al. [21] respectively adopted LR, RF, SVM, and NB to predict drug repositioning candidates. Moreover, Ozsoy et al. [22] developed a recommendation system based on Pareto dominance and collaborative filtering to identifying drug-disease associations. Diseases shared among neighbour drugs were used to infer potentials and novel indications for existing drugs. Furthermore, Luo et al. [23] introduced a Drug Repositioning Recommendation System (DRRS) based on a singular value thresholding algorithm for identifying drug repositioning candidates in heterogeneous drug-disease networks. More recently, Liu et al. [24], Zhang et al. [25], Xuan et al. [26], and Zhang et al. [27] introduced NRLMF, CMFDD, DisDrugPred, DRIMC to identify drug candidate therapeutic indications. However, these models have not fully exploited the widely available drug and disease data sources. Moreover, most of these approaches have considered drug and disease similarity information without tackling data noise and redundancy issues that may have a substantial effect on the prediction accuracy. Furthermore, these models have not overcome the issue of highly skewed gold-standard drug-disease interaction information.

In this study, an integrative framework, SNF-NN, which is using similarity measures, similarity selection, Similarity Network Fusion (SNF), and a Neural Network (NN) deep learning model to improve the drug-disease interaction prediction accuracy and predict novel drug-disease interactions. The prediction of new interactions is performed using known drug-disease interactions and very informative heterogeneous similarity information generated from different drug-related and disease-related information datasets. The materials section discusses the datasets used in this study. The parts of SNF-NN are described in the methods section. In the results and discussion section, the robustness of the proposed is verified by comparing its performance with baseline as well as state-of-the-art machine learning models. To further demonstrate SNF-NN’s efficiency in predicting novel drug-disease interactions, predicted unknown drug-disease interactions are validated against clinical trials and published studies.

## Methods

Three benchmark datasets were used to verify the robustness of SNF-NN. Each benchmark dataset consisted of known drug-disease interactions, drug-related similarity information types as well as disease-related similarity information types. The first benchmark dataset, termed SND, was assembled and used along with two datasets, i.e. Cdataset [17] and LRSSL [28], which were used in previous studies [17, 23, 26–28].

Herein, drug-disease interactions in each dataset were represented by a binary matrix $Y \in R^{\; m \times n}$, where *m* and *n* are the number of drugs and the number of diseases retrieved in the specific dataset, respectively. An entry $y_{ij} \in {0,1}$ was set to 1 if the drug $r_i$ is therapeutically used to treat the indicated disease $d_j$, otherwise $y_{ij}$ was set to 0 if the interaction between $r_i$ and $d_j$ was unknown.

Drug-related similarity types in each dataset were represented by a set of binary adjacency matrices *SR*, where each binary adjacency matrix $sr \in R^{\; m \times m}$, and *m* is the distinct number of drugs across all drug-related similarity types. The value of the entry $sr_{ij} \in [0,1]$ quantifies the similarity between drugs $r_i$ and $r_j$, where 1 demonstrates perfect similarity, and 0 demonstrates absolute dissimilarly. To handle drug synonyms within the same dataset and/or across different datasets, drugs chemical, generic, or commercial names were mapped to their DrugBank’s Accession Numbers (DBANs). This mapping is required to merge records of drugs with synonyms.

Disease-related similarity types in each dataset were represented by a set of binary adjacency matrices *SD*, where each binary adjacency matrix $sd \in R^{\; n \times n}$, and *n* is the distinct number of diseases across all disease-related similarity types. The value of the entry $sd_{ij} \in [0,1]$ quantifies the similarity between diseases $d_i$ and $d_j$, where 1 demonstrates perfect similarity, and 0 demonstrates absolute dissimilarly. To handle disease synonyms, disease names were mapped to their UMLS’s Concept Unique Identifier (CUI), and records of diseases with synonyms were merged.

Further details about the benchmark datasets are presented in Table 1.

**Table 1:** Details of benchmark datasets

Benchmark dataset	No. drugs	No. disease	No. interactions	Sparsity	No. drug similarities (%)	No. disease similarities
SND	867	803	8684	98.75	10	14
Cdataset [17]	593	313	1933	98.96	1	1
LRSSL [28]	763	681	3051	99.41	3	1

### SND benchmark dataset

The SND benchmark dataset was assembled from various biological and biomedical data sources. It consists of three different types of information, namely drug-disease interaction data, drug-related similarity data, and disease-related similarity data.

Drug-disease interaction data (i.e., gold-standard data) was collected from two widely used data sources, namely DrugBank [29] and repoDB [30]. The gold-standard data consists of 867 FDA-approved drugs, 803 diseases, and 8684 clinically reported and/or experimentally validated drug-disease interactions with 98.75% sparsity.

Drug-related similarity data has 10 networks based on: (1) target protein interactions, (2) side effects, (3) chemical structures, (4) GO molecular functions, (5) GO biological processes, (6) GO cellular components, (7) metabolism enzymes, (8) protein sequences, (9) anatomical therapeutic chemical classification codes, and (10) drug pairwise interactions.

Disease-related similarity data has 14 networks based on: (1) curated genes, (2) HPO genes, (3) literature-based genes, (4) curated variants, (5) literature-based variants, (6) microRNAs, (7) long non-coding RNAs, (8) HPO phenotypes, (9) IS-A taxonomy, (10) information-theoretic similarity, (11) GO terms, (12) implicit semantic similarity, (13) semantic and gene functional, and (14) curated association type ontology.

Further details about drug-related and disease-related similarity data are provided in the Additional file 1. All similarity and interaction matrices are provided in http://pages.cpsc.ucalgary.ca/ tnjarada/snf-nn.php.

### Cdataset benchmark dataset

The Cdataset benchmark dataset was retrieved from a previous study [17]. It consists of three different types of information, namely drug-disease interaction data, drug-related similarity data, and disease-related similarity data.

Drug-disease interaction data includes 593 FDA-approved drugs listed in the DrugBank Database [29], 313 diseases registered in the Online Mendelian Inheritance in Man (OMIM), and 1933 known drug-disease interactions with 98.96% sparsity.

Drug-related similarity data includes one network based on chemical structures. The canonical Simplified Molecular Input Line Entry System (SMILES) [31] were extracted from the DrugBank Database [29]. Drug pairwise similarity was calculated using the CDK [32] and the Tanimoto score [33] of the 2-D chemical fingerprints.

Disease-related similarity data has one network based on human phenotypes that were obtained from the OMIM Database [34]. Disease pairwise similarity was computed using MeSH terms [35] and the semantic similarity measure introduced in [36].

### LRSSL benchmark dataset

The LRSSL benchmark dataset was obtained from the paper [28]. It consists of three different types of information, namely drug-disease interaction data, drug-related similarity data, and disease-related similarity data.

Drug-disease interaction data has 3051 between 763 FDA-approved drugs and 681 diseases with 99.41% sparsity.

Drug-related similarity data contains three networks based on (1) chemical structures, (2) target protein domains, and (3) GO target protein annotations. Drug chemical fingerprints of the drugs were retrieved from the PubChem Database [37]. Protein domains of drug target proteins were extracted from the InterPro Protein Families Database [38]. GO’s molecular function and biological process terms of target proteins were extracted from the UniProt Database [39].

Disease-related similarity data includes one network based on human phenotypes that were retrieved from the OMIM Database [34]. Disease pairwise similarity was calculated using the MimMiner semantic similarity measure [36].

## Methods

### Problem description

A set of drugs $R = {r_1,r_2,\ldots ,r_m}$ and a set of diseases $D = {d_1,d_2,\ldots ,d_n}$ are defined in which *m* and *n* are the numbers of drugs in *R* and diseases in *D*, respectively. For *R*, we define a set of similarity adjacency matrices between drugs based on different drug-related datasets as *SR*, where $SR = {sr_1,sr_2,\ldots ,sr_k}$ and *k* is the number of drug-related datasets. The dimension of each drug similarity adjacency matrix is $m \times m$; where *m* is number of distinct drugs across the *k* drug-related datasets and $sr_a(r_u,r_w)$ indicates how much the pair of drugs $r_u$ and $r_w$ are alike based on drug-related dataset *a*. All of the values in the *SR* similarity adjacency matrices are in the range of [0, 1], where 0 indicates absolute dissimilarly, and 1 indicates perfect similarity between a pair of drugs. Similarly, let a set of similarity adjacency matrices between diseases, *D*, be defined based on different disease-related datasets as *SD*, where $SD = {sd_1,sd_2,\ldots ,sd_l}$ and where *l* is the number of disease-related datasets. The dimension of each disease similarity adjacency matrix is $n \times n$; where *n* is the number of distinct diseases across the *l* disease-related datasets. The value of $sd_b(d_x,d_z)$ indicates how much the pair of disease $d_x$ and $d_z$ are alike based on the disease-related dataset *b*. All values in the *SD* similarity adjacency matrices are in the range of [0, 1], where 0 indicates absolute dissimilarly, and 1 indicates perfect similarity between a pair of diseases. Next, the interactions between the set of drugs *R* and the set of diseases *D* were defined as a binary matrix *Y* of dimension $m \times n$ in which $y_{ij}= 1$ if drug $r_i$ interacts with disease $d_j$, and $y_{ij} = 0$ if there is no known evidence that drug $r_i$ interacts with disease $d_j$. Given the matrix *Y*, and the two sets of matrices *SR* and *SD*, the aim is to predict novel drug-disease interactions in *Y*.

### Description of the SNF-NN method

The SNF-NN method is presented as a novel approach that offers insight into drug-disease interaction discovery by leveraging drug-related similarity information, disease-related similarity information, and known drug-disease interactions. SNF-NN integrates similarity measures, similarity selection, Similarity Network Fusion (SNF), and Neural Network (NN) and performs a non-linear analysis that improves the drug-disease interaction prediction accuracy.

**Fig. 1:**
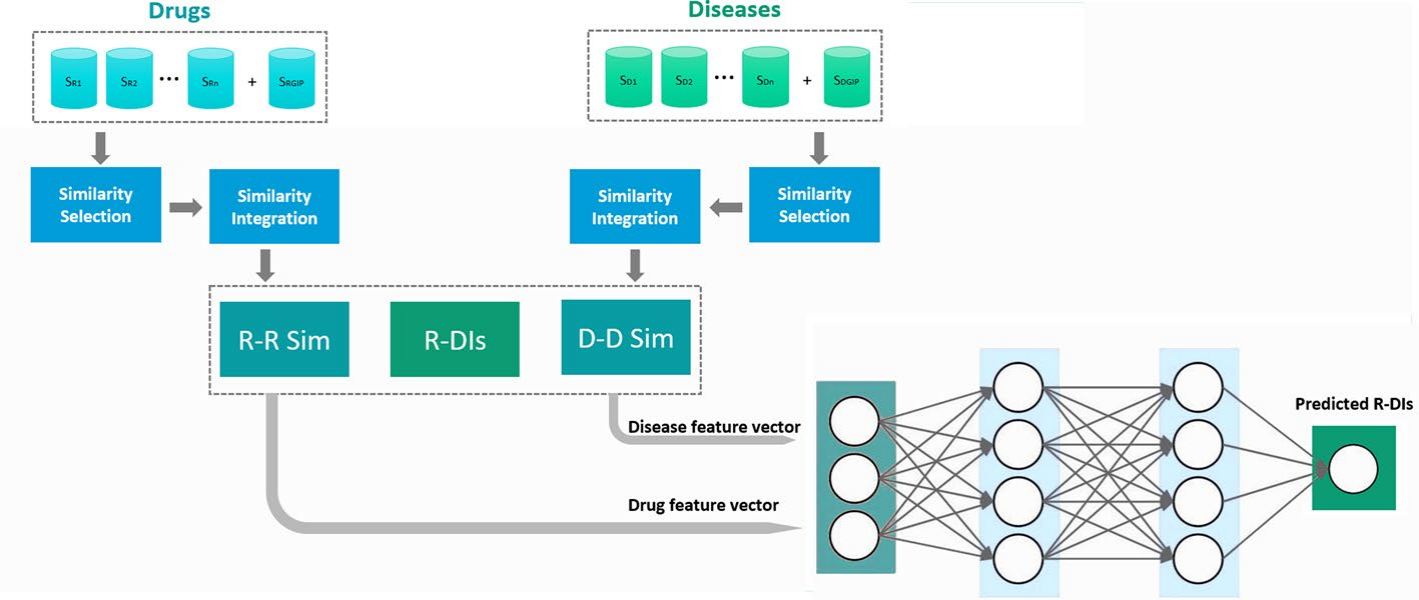
The overall workflow of the SNF-NN approach

The SNF-NN method consists of four steps. In the first step, it acquires drug-related information (e.g., drug-target proteins, drug-chemical structures, drug-side effects), disease-related information (e.g., disease-gene associations, disease-miRNA associations, disease phenotypes), and known drug-disease interactions information from various sources. Next, it leverages the most literature-recognized similarity measure for each drug-related or disease-related information type to calculate pairwise drug or disease similarity for that specific drug-related or disease-related information type. Furthermore, it calculates the Gaussian Interaction Profile (GIP) similarity for drug pairs and disease pairs based on the known drug-disease interactions. In the second step, it uses a heuristic process to select the most insightful and less redundant subset of drug and disease similarity types. In the third step, it utilizes a non-linear similarity network fusion method to integrate the vetted drug and disease similarity types. In the fourth step, it initially performs a Cartesian product on the fused drug and disease similarity information concatenating the feature vectors of each drug-disease pair. Finally, it feeds the concatenated feature vectors and known drug-disease interactions to a multi-layer neural network to predict novel interactions between input drug-disease pairs. The overall workflow of the SNF-NN approach is shown in Fig. 1.

### Calculating similarity measures

Pairwise similarity is calculated for each drug-related and disease-related dataset in order to quantify the shared characteristics between each drug pair or disease pair. The most literature-recognized similarity measure for each drug-related and disease-related information type is adopted. Drug and disease pairwise similarity values are in the range [0, 1] between a pair of drugs or diseases, where 0 represents the least similarity, and 1 represents the most similarity. Furthermore, it is assumed that a pair of drugs that interact with diseases in known drug-disease interactions will also behave similarly to new diseases. Similarly, it is assumed that a pair of diseases that interact with drugs in known drug-disease interactions will behave the same way when they are cured by new drugs. Therefore, the drug-disease interaction information is used to calculate the Gaussian interaction profile kernel [40] as profile similarity between drug pairs or disease pairs.

Each drug *r* in a drug-disease interactions dataset is represented by a binary feature vector of the diseases in the dataset *f*(*r*), where the absence or presence of a disease interaction is encoded by 0 or 1, respectively. Similarly, each disease *d* in the drug-disease interactions dataset is represented by a binary feature vector of the drugs in the dataset *g*(*d*), where the absence or presence of a drug interaction is encoded by 0 or 1, respectively. The pairwise drug and disease profile similarity values are normalized by dividing by the average number of disease interactions per drug and drug interactions per disease, respectively.

$$\begin{aligned}&sr_{(GIP)} (r_a,r_b)= exp\Big (\frac{-\tau m {||f(r_a)-f(r_b)||}^2}{\sum _{i=1}^{m}{{|f(r_i)|}^2}}\Big ), \\&sd_{(GIP)} (d_a,d_b)= exp\Big (\frac{-\tau n {||g(d_a)-g(d_b)||}^2}{\sum _{j=1}^{n}{{|g(d_j)|}^2}}\Big ) \end{aligned}$$

where the parameter $\tau$ controls the kernel bandwidth, *m* and *n* are the total number of drugs and diseases in the drug-disease interactions dataset, $|f(r_i)|$ is the number of disease interactions for drug $r_i$, $|g(d_j)|$ is the number of drug interactions for disease $d_j$. Here, $\tau$ was simply set to 1 as indicated by van Laarhoven et al. [40].

### Similarity selection

SNF-NN applies to any set of drug-related and disease-related similarity types. However, the quality, richness, and correlation of such drug-related and disease-related similarity types are essential when trying to integrate these different similarity types. Data inconsistency and redundancy may lead to noise in the integrated drug-related similarity matrix. Thus, we use an effective method, introduced by Olayan et al. [41], to select the most informative, most insightful, and less redundant subset combination of drug similarity measures. This heuristic similarity selection process involves four steps and explained in [42].

### Similarity network fusion

The objective of this step is to integrate the highly informative and less redundant similarity matrices from the previous step into one comprehensive drug similarity matrix that captures the shared information and any complementary knowledge from across the set of similarity matrices. Therefore, given a set of drug similarity matrices, we construct a fused similarity matrix using the similarity network fusion approach introduced by Wang et al. [43]. Given a set of drug similarity matrices, the SNF approach uses an iterative non-linear process based on message-passing theory to consolidate the given set into one comprehensive matrix. SNF iteratively applies the K-Nearest Neighbors (KNN) algorithm to update each drug similarity matrix with information from the other drug similarity matrices until a drug similarity matrix that represents the initial set of drug similarity matrices as good as possible is obtained.

Given the subsets of drug-related and disease-related similarity matrices selected in the previous step, the goal of this step is to integrate these selected similarity matrices into two comprehensive similarity matrices for drugs and diseases. Therefore, given a set of multiple similarities matrices of drugs and diseases, respectively, the iterative non-linear similarity network fusion process is applied to construct two fused drug-drug and disease-disease similarity matrices, separately. These two fused similarity matrices capture the shared information and any complementary knowledge from across the similarity matrices of drugs and diseases, respectively.

### Neural network model

The neural network structure definition can have a significant impact on the prediction performance of the neural network model. As an effective neural network structure, the feed-forward multi-layer perceptron network type has recently received extensive attention in the field of computational biology. The information in such a neural network can only move in one direction, forward, from the input layer, through the hidden layer(s) to the output layer; hence, there are no cycles or loops in the network. The input layer contains the initial data for the neural network; hidden layers are intermediate layers where all the computations happen, and the output layer produces the result for the given data input. Each layer in a neural network is made up of a number of computational neurons or units. A neuron is connected to a set of neurons in the next layer. Each neuron has a weight value, which can be defined as the impact of the neuron on the next layer,s neurons. Each neuron’s input value gets multiplied by the neuron’s weight to get the neuron’s output value that gets passed to the next layer. The performance and speed of learning of a neural network model are highly dependent on the number of hidden layers, along with the number of neurons in each hidden layer. Herein, a fully connected feed-forward multi-layer perceptron network model is introduced to further improve the accuracy of predicting drug-disease interactions in a deep-learning manner.

In order to achieve the best overall accuracy, a tedious process is performed to tune various hyperparameters of the deep neural network model. Practically speaking, the number of hidden layers, the number of neurons in each layer, the type of activation function applied behind each layer, and the learning dropout rate in each layer [44] should be specified. In addition, other hyperparameters ranging from the $\alpha$, $\beta _1$, $\beta _2$, and $\epsilon$ for the Adam Optimization Algorithm [45] to the weight and bias parameters [46], the number of epochs, and the batch size should possibly be examined.

It is worth mentioning that the importance of each hyperparameter depends on the trained data, and some hyperparameters might be more important than others. For instance, fiddling around with the number of hidden layers, the number of neurons in each layer, or the learning rate would sometimes make a huge difference.

To decide on the neural network architecture, nested cross-validation [47] is used to systematically organize the hyperparameters tuning process and make it more efficient while converging to a suitable hyperparameters setting. Applying the hyperparameters tuning process without using nested cross-validation would cause model overfitting because the same data is used both to tune and to evaluate model performance. The hyperparameters in a nested cross-validation are tuned as follows:

1. The model hyperparameters are set to certain values.
2. The input dataset is split into three folds.
3. The model is trained using the current hyperparameter values and two dataset folds.
4. The model is tested using the chosen hyperparameter values and the remaining dataset fold (test set).
5. Step 3 and 4 are repeated until each dataset fold is considered as the test set.
6. The model performance results and the combination of hyperparameter values are recorded.
7. Steps 1 to 6 are carried out for all combinations of hyperparameter values.
8. The combination of hyperparameter values leads to the best overall performance is selected.

The following hyperparameters and associated values are used to decide on the neural network model architecture:

- Number of hidden layers: {1, 2, 3, 4, 5}
- Number of neurons in each hidden layer: {100, 200, 300, 400, 500}
- Activation functions:
  - Logistic sigmoid $$\sigma (z)= \frac{1}{1+e^{-z}}$$
  - Hyperbolic tangent $$TanH(z)= \frac{sinh(z)}{cosh(z)} = \frac{e^zz-e^{-z}}{e^z+e^{-z}}$$
  - Rectified linear unit $ReLU(z)=max(0,z)$
- Dropout rate: {0.3, 0.35, 0.4., 0.45, 0.5}

The best performance is achieved with a deep neural network model of 4 hidden layers with 300 neurons and a Dropout rate of 0.35 for each layer. The rectified linear unit activation function is applied to all neurons in all hidden layers. As predicting drug-disease interactions is a binary classification problem, the logistic sigmoid activation function is applied to the output layer, and the binary cross-entropy loss function is used to calculate loss values.

Moreover, the deep neural network model yields the best results when He Initialization [48] is used to initialize weight and bias parameters and Adam Optimization Algorithm to update these parameters. As recommended in [45], Adam’s hyperparameters were set as follows:

- The learning rate $(\alpha )$: 1E−3.
- The exponential decay rate for the first-moment estimates $(\beta _1)$: 0.9
- The exponential decay rate for the second-moment estimates $(\beta _2)$: 0.999
- The small constant for numerical stability $(\epsilon )$: 1E−7

Finally, the model is fed batch inputs with a batch size of 100. The number of epochs is set to 100 for the different datasets. All trained deep neural network models with their hyperparameters settings and performance in nested cross-validation results are presented in the Additional file 1.

## Results and discussion

### Datasets

To evaluate SNF-NN, a new benchmark dataset, SND, was assembled from various resources. Furthermore, two benchmark datasets, namely Cdataset [17] and LRSSL [28], were downloaded to validate the proposed method. Cdataset and LRSSL datasets were used to evaluate state-of-the-art computational drug repositioning methods [17, 23, 26–28]. Each dataset is a heterogeneous network consisting of known drug-disease interactions, drug similarity network(s), disease similarity network(s). The statistics of the datasets used are reported in Table 1.

### Evaluation criteria

Herein, the systematic evaluation criteria for verifying the robustness and predictive performance of SNF-NN is described. First, stratified 10-fold cross-validation was performed, and each gold-standard of the SND, Cdataset, and LRSSL datasets was split into training and test sets to prevent any over-optimistic evaluation of the model performance. During the stratified 10-fold cross-validation, the known drug-disease interactions (i.e., positive relations) with a matching number of unknown drug-disease interactions (i.e., negative relations) were randomly split into ten sets. Each set has equal numbers of positive and negative relations. In each cross-validation trial, nine sets were taken in turn as the training set, while the remaining set represents the test set. The 10-fold cross-validation was repeated five times with different random seed values, and the average performance was calculated in order to avoid any high-variance and biased cross-validation evaluation.

In practice, a lot is learned about deep neural network models when observing their performance during the learning/training process. For each epoch, model training metrics (i.e., accuracy and loss) are recorded and two plots are created to describe the learning process of the model trained. These two plots provide insight into the model’s learning process, such as its speed of convergence over epochs and whether it may have already converged or not.

Subsequently, the confusion matrix evaluation metrics are calculated. These metrics are true positive (TP), true negative (TN), false positive (FP), and false negative (FN). To put things into perspective, TP represents the known RDIs that are correctly predicted, while TN represents the unknown RDIs that are correctly predicted by the model. However, FP represents the unknown RDIs that are predicted, and FN represents the known RDIs that are not predicted by the model. Accuracy (*Acc*), Specificity (*Spec*), Precision (*Prec*), Recall (*Rec*), and F1-score (*F*1) evaluation metrics are calculated for the performance comparison.

$$\begin{aligned}&Acc= \frac{TP+TN}{TP+TN+FP+FN} \\&Spec= \frac{TN}{TN + FP} \\&Prec= \frac{TP}{TP + FP} \\&Rec= \frac{TP}{TP + FN} \\&F1= 2 \times \frac{Precision \times Recall}{Precision + Recall} \end{aligned}$$

Moreover, the Matthews Correlation Coefficient (*MCC*) is adopted to evaluate the proposed model. *MCC* is widely used in the field of bioinformatics for binary classification machine learning models [49]. The *MCC* is defined as follows:

$$\begin{aligned} MCC= \frac{TP \times TN - FP \times FN}{\sqrt{(TP+FP)(TP+FN)(TN+FP)(TN+FN)}} \end{aligned}$$

Furthermore, the Area Under Curve - Receiver Operator Characteristic ($AUC-ROC$) and the Area Under Curve - Precision-Recall ($AUC-PR$) are used as the primary performance evaluation metrics. $AUC-ROC$ shows the trade-off between the true positive (i.e., the correctly predicted known RDIs) and the false positive (i.e., the wrongly predicted unknown RDIs) which eventually gives the model a successful classification rate. However, $AUC-PR$ gives a more informative picture of model performance when dealing with highly skewed gold-standard datasets of more than 98.5% sparsity [50].

The three benchmark datasets (i.e., SND, Cdataset, LRSSL) and the above eight evaluation metrics (i.e., *Acc*, *Spec*, *Prec*, *Rec*, *F*1, *MCC*,$AUC-ROC$, $AUC-PR$) are utilized to evaluate various aspects of the performance of the SNF-NN model.

The effectiveness of the SNF-NN model is validated by comparing its performance with nine baseline machine learning classification models, namely, Stagewise Additive Multi-class Modeling Exponential (SAMME) loss function [51], Decision Tree (DT) [52], Gaussian Process Classification (GPC) based on Laplace approximation [53], K-Nearest Neighbors (KNN) [54], Gaussian Naïve Bayes (GNB) [55], Quadratic Discriminant Analysis (QDA) [56], Random Forest (RF) [57], Linear Support Vector Machine (SVM), and Radial Basis Function (RBF) SVM [58]. To have a fair comparison, the similarity selection and fusion described in the previous section are applied to all drug-related and disease-related similarity information types and the integrated drug and disease similarity matrices are used as inputs for the baseline methods. Notably, the parameters of these models have a great impact on their performance. Hence, the parameters for each model are tuned and the best performance is reported. The comparison with the nine baseline models is conducted using all the benchmark datasets and evaluation metrics.

The robustness of the SNF-NN method is verified by comparing its performance with five state-of-the-art machine learning methods for drug-disease interaction prediction, namely, MBiRW [17], SCMFDD [25], DRRS [23], DisDrugPred [26], and DRIMC [27]. Additionally, two state-of-the-art matrix factorization based methods for drug-disease interaction prediction (i.e., KBMF [59] and NRLMF [24]) are also included in the comparison. It is noteworthy that the seven state-of-the-art models were previously evaluated using the Cdataset and LRSSL benchmark datasets [27]. Hence, the same benchmark datasets are used to evaluate the SNF-NN against the seven state-of-the-art methods fairly. The $AUC-ROC$ and $AUC-PR$ are used as evaluation metrics.

Finally, the abilities of the SNF-NN method to predict novel drug-disease interactions is verified by validating the predicted unknown drug-disease interactions (i.e., false positive interactions) against up-to-date clinical trial studies and pharmaceutical indications resources such as ClinicalTrials.gov [60], DrugBank [29], and PubMed [61].

### Evaluation results

#### SNF-NN performance during the training process

The performance of SNF-NN during the training process is captured using the accuracy and loss metrics with two plots for each of the three benchmark datasets for the training set over the training epochs.

**Fig. 2:**
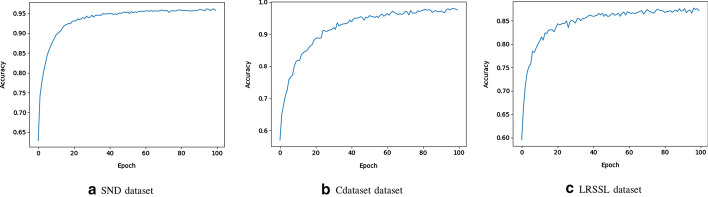
SNF-NN accuracy on the training set of SND, Cdataset, and LRSSL datasets

Figure 2 shows model accuracy on the training set of SND, Cdataset, and LRSSL benchmark datasets, respectively. It is noticed that the trend for accuracy on all benchmark datasets jumps to more than 0.85 during the first 20 epochs, meaning the deep neural network model is learning quickly. Moreover, the accuracy trend appears to be steady in the last epochs, which means that the number of epochs selected is appropriate, and that the SNF-NN method can no longer be trained.

**Fig. 3:**
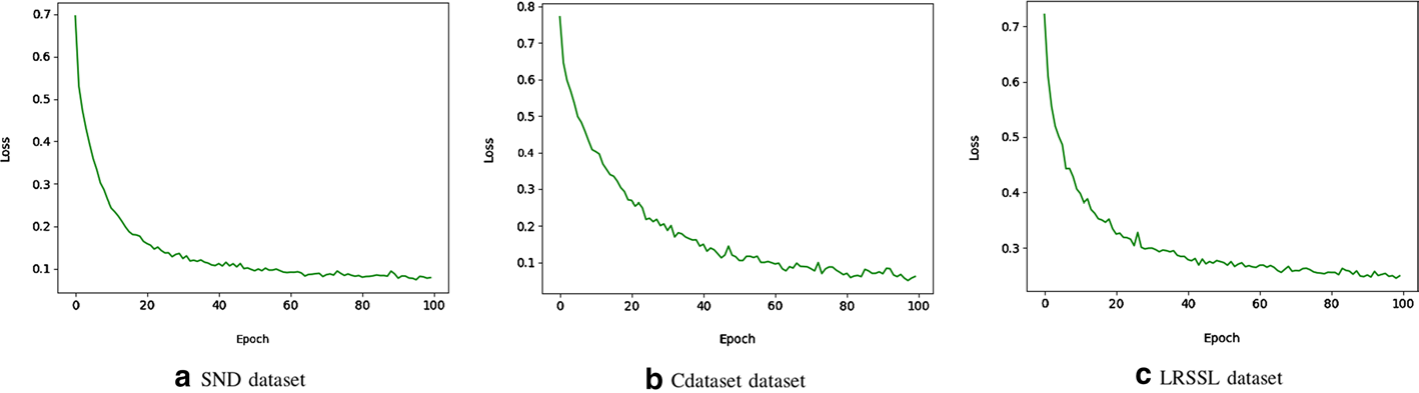
SNF-NN loss on the training set of SND, Cdataset, and LRSSL datasets

Figure 3 shows model loss on the training set of SND, Cdataset, and LRSSL benchmark datasets, respectively. It is noticed that the trend for loss on all benchmark datasets decreases rapidly, which means that the deep neural network model approaches its local minimum quickly. Furthermore, the loss trend is steady for the last epochs, meaning the number of epochs selected is appropriate, and that the SNF-NN method can not be trained any further.

#### Comparison of SNF-NN performance with baseline machine learning models

The overall reliability and effectiveness of SNF-NN are investigated by evaluating its performance against nine widely used baseline machine learning models (i.e., SAMME, DT, GPC, KNN, GNB, QDA, RF, Linear-SVM, RBF-SVM). The comparison of SNF-NN performance with the baseline methods is carried out using stratified 10-fold cross-validation on the three benchmark datasets (i.e., SND, Cdataset, LRSSL). The comparison results based on all evaluation metrics defined previously are reported in Tables 2, 3, and 4. The similarity selection and fusion described in the previous section are applied to drug-related and disease-related similarity information types in each benchmark dataset and the fused similarity matrices are used as inputs for the baseline methods.

As shown in Table 2, SNF-NN outperforms all the baseline methods on the SND benchmark dataset with a substantial difference in terms of most of evaluation metrics (*Acc* = 0.796, *Rec*= 0.816, *F*1 = 0.800, *MCC* = 0.593, $AUC-ROC$ = 0.867, and $AUC-PR$ = 0.876).

Moreover, the results in Table 3 demonstrates the superiority of SNF-NN performance over the all the machine learning methods on the Cdataset benchmark dataset with a considerable variance with respect to most of evaluation metrics (*Acc* = 0.783, *F*1 = 0.790, *MCC* = 0.569, $AUC-ROC$ = 0.879, and $AUC-PR$ = 0.856).

Furthermore, Table 4 verifies the the overall performance of the proposed method on the LRSSL benchmark dataset with a significant difference in terms of almost all of the evaluation metrics (*Acc* = 0.846, *Prec* = 0.821, *Rec*= 0.793, *F*1 = 0.807, *MCC* = 0.617, $AUC-ROC$ = 0.936, and $AUC-PR$ = 0.903).

While the *Spec* metric is defined to evaluate the performance of machine learning methods in predicting negative relations (i.e., no RDIs), SNF-NN focuses on predicting positive relations (i.e., novel RDIs). Therefore, it is not significant that the SNF-NN does not have the highest performance in terms of the *Spec* evaluation metric.

Finally, it is worth emphasizing that the *F*1 metric is defined as the harmonic mean of *Prec* and *Rec*; thus, it is not surprising that the SNF-NN does not have the highest performance with respect to *Prec* and *Rec* when it has the best *F*1 value.

**Table 2:** SNF-NN performance comparison with baseline machine learning methods on the SND benchmark dataset

Method	Accuracy	Specificity	Precision	Recall	F1-score	MCC	AUC-ROC	AUC-PR
SAMME	0.682	0.841	0.767	0.523	0.621	0.384	0.682	0.764
DT	0.553	**0.985**	**0.888**	0.122	0.214	0.211	0.553	0.725
GPC	0.646	0.676	0.655	0.615	0.634	0.292	0.646	0.731
KNN	0.650	0.619	0.641	0.681	0.661	0.300	0.650	0.741
GNB	0.669	0.586	0.645	0.751	0.694	0.342	0.669	0.760
QDA	0.649	0.632	0.646	0.666	0.654	0.300	0.649	0.740
RF	0.533	0.692	0.635	0.374	0.354	0.112	0.533	0.661
Linear-SVM	0.702	0.718	0.709	0.685	0.697	0.404	0.702	0.776
RBF-SVM	0.535	0.949	0.704	0.120	0.204	0.124	0.535	0.632
SNF-NN	**0.796**	0.777	0.785	**0.816**	**0.800**	**0.593**	**0.867**	**0.876**

The best value of each evaluation metric is shown in bold

**Table 3:** SNF-NN performance comparison with baseline machine learning methods on the Cdataset benchmark dataset

Method	Accuracy	Specificity	Precision	Recall	F1-score	MCC	AUC-ROC	AUC-PR
SAMME	0.666	0.683	0.672	0.649	0.660	0.333	0.666	0.748
DT	0.611	0.809	0.711	0.412	0.505	0.253	0.610	0.709
GPC	0.707	0.604	0.692	0.811	0.738	0.433	0.707	0.799
KNN	0.695	0.560	0.654	**0.830**	0.731	0.406	0.695	0.785
GNB	0.654	0.645	0.651	0.662	0.656	0.308	0.654	0.741
QDA	0.629	0.472	0.599	0.786	0.679	0.272	0.629	0.746
RF	0.618	0.612	0.618	0.624	0.620	0.237	0.618	0.715
Linear-SVM	0.692	0.673	0.685	0.712	0.698	0.386	0.692	0.771
RBF-SVM	0.530	**1.000**	**0.994**	0.060	0.112	0.172	0.530	0.762
SNF-NN	**0.783**	0.754	0.769	0.813	**0.790**	**0.569**	**0.879**	**0.856**

The best value of each evaluation metric is shown in bold

**Table 4:** SNF-NN performance comparison with baseline machine learning methods on the LRSSL benchmark dataset

Method	Accuracy	Specificity	Precision	Recall	F1-score	MCC	AUC-ROC	AUC-PR
SAMME	0.665	0.705	0.680	0.624	0.650	0.331	0.665	0.746
DT	0.635	0.775	0.689	0.494	0.574	0.282	0.635	0.718
GPC	0.701	0.717	0.709	0.685	0.696	0.403	0.701	0.776
KNN	0.661	0.553	0.633	0.769	0.694	0.329	0.661	0.759
GNB	0.616	0.509	0.596	0.723	0.653	0.238	0.616	0.729
QDA	0.567	0.662	0.613	0.473	0.453	0.161	0.567	0.675
RF	0.611	0.683	0.631	0.539	0.580	0.225	0.611	0.700
Linear-SVM	0.678	0.672	0.676	0.685	0.680	0.357	0.678	0.759
RBF-SVM	0.578	**0.869**	0.687	0.286	0.403	0.191	0.578	0.665
SNF-NN	**0.846**	0.780	**0.821**	**0.793**	**0.807**	**0.617**	**0.936**	**0.903**

The best value of each evaluation metric is shown in bold

#### Comparison of SNF-NN performance with state-of-the-art drug-disease interaction prediction models

The robustness of SNF-NN is verified by evaluating its performance against seven state-of-the-art models for drug-disease interaction prediction, namely, KBMF [59], NRLMF [24], MBiRW [17], DRRS [23], SCMFDD [25], DisDrugPred [26], and DRIMC [27]. The comparison of SNF-NN performance with state-of-the-art models is conducted using stratified 10-fold cross-validation on the Cdataset and LRSSL benchmark datasets. Herein, it is important to highlight that the seven state-of-the-art models were previously evaluated using both Cdataset and LRSSL benchmark datasets and the the $AUC-ROC$ and $AUC-PR$ evaluation metrics [27]. The comparison results are reported in Tables 5 and 6.

As shown in Table 5, SNF-NN surpasses all the seven state-of-the-art models on the Cdataset benchmark dataset ($AUC-ROC$ = 0.879, and $AUC-PR$ = 0.856) with a significant difference of 55.5% in terms of $AUC-PR$ metric. $AUC-PR$ is a powerful and informative metric when dealing with highly skewed datasets [50].

Furthermore, Table 6 confirms the robustness of the proposed method by validating its performance on the LRSSL benchmark dataset. SNF-NN achieves superior performance by improving the $AUC-ROC$ by 2.8%, and $AUC-PR$ by 56.7%, which indicates the reliability and effectiveness of SNF-NN in predicting novel drug-disease interactions in highly skewed gold-standard datasets.

**Table 5:** SNF-NN performance comparison with state-of-the-art models for drug-disease interaction prediction on the Cdataset benchmark dataset

Year	Method	AUC-ROC	AUC-PR
2013	KBMF [59]	0.754	0.134
2016	NRLMF [24]	0.850	0.315
2016	MBiRW [17]	0.813	0.149
2018	DRRS [23]	0.783	0.113
2018	SCMFDD [25]	0.749	0.044
2019	DisDrugPred [26]	0.846	0.234
2020	DRIMC [27]	0.878	0.301
2020	SNF-NN	**0.879**	**0.856**

The best value of each evaluation metric is shown in bold

**Table 6:** SNF-NN performance comparison with state-of-the-art models for drug-disease interaction prediction on the LRSSL benchmark dataset

Year	Method	AUC-ROC	AUC-PR
2013	KBMF [59]	0.648	0.130
2016	NRLMF [24]	0.872	0.326
2016	MBiRW [17]	0.816	0.167
2018	DRRS [23]	0.794	0.073
2018	SCMFDD [25]	0.724	0.032
2019	DisDrugPred [26]	0.872	0.278
2020	DRIMC [27]	0.908	0.336
2020	SNF-NN	**0.936**	**0.903**

The best value of each evaluation metric is shown in bold

#### Efficiency of SNF-NN to predict novel drug-disease interactions (repositioning candidates)

While it is essential that computational models correctly predict known drug-disease interactions, the most important task is to identify novel drug-disease interactions, or false positives, as repositioning candidates.

To further investigate the reliability of the proposed method, a randomly selected set of five thousand unknown drug-disease interactions of the SND dataset is fed to the SNF-NN model. The SNF-NN model predicts 680 novel drug-disease interactions related to 350 drugs, in which a minimum of five new therapeutic uses are associated with 24 drugs and one novel therapeutic use is associated with 194 drugs.

The novel drug-disease interactions of the top five drugs are validated against approved clinical trial studies in ClinicalTrials.gov, DrugBank, or PubMed, and substantial evidence is discovered. Among the top 34 predicted drug-disease interactions, 25 (73.5%) interactions are actually valid, but they are not annotated in the SND benchmark dataset.

For instance, the interaction between *Loteprednol* drug and *Dry Eye Syndromes* is not annotated in the SND gold-standard dataset while the proposed model predicts it. Another example is the interaction between *Sparfloxacin* drug and *Pneumonia Due to Klebsiella Pneumoniae* disease, which is also successfully identified by the SNF-NN model. Table 7 presents the novel drug-disease interactions of the top five drugs predicted by SNF-NN along with their source of evidence. Furthermore, a list of all the 680 novel drug-disease interactions predicted by SNF-NN is reported in Additional file 1.

**Table 7:** The novel drug-disease interactions of the top five drugs predicted by SNF-NN along with their source of evidence, where a hyphen indicates a previously unknown drug-disease interaction

Drug name	Disease name	Evidence
Dexamethasone	Endometriosis	ClinicalTrials.gov
	Hereditary Orotic Aciduria	PubMed
	Hepatitis C, Chronic	PubMed
	Mineral Deficiency	DrugBank.ca
	Urinary Tract Infection	ClinicalTrials.gov
	Q Fever Endocarditis	PubMed
	Hepatolenticular Degeneration	–
Levofloxacin	Vomiting	PubMed
	Mixed Anxiety and Depressive Disorder	PubMed
	Lymphogranuloma Venereum	PubMed
	Echinococcus Granulosus Infection of Liver	PubMed
	Schistosomiasis	PubMed
	Malignant Neoplasm of Stomach Stage IV	–
	Scurfiness of Scalp	–
Loteprednol	Streptococcal Pneumonia	PubMed
	Dry Eye Syndromes	ClinicalTrials.gov
	Tinea Cruris	–
	Sepsis Due to Staphylococcus Aureus	–
	Drug-Induced Mucositis	–
	Yaws	–
	Relapsing Fever	–
Sparfloxacin	Pneumonia due to Klebsiella Pneumoniae	DrugBank.ca
	Urinary Retention	PubMed
	Intractable Hiccups	–
	Salmonella Sepsis	–
	Osteoporosis, Postmenopausal	–
	Dacryocystitis	–
	Uric Acid Renal Calculus	–
Triamcinolone	Impetigo	PubMed
	Osteoporosis	PubMed
	Coughing	ClinicalTrials.gov
	Obesity	ClinicalTrials.gov
	Multiple Myeloma	PubMed
	Gonococcal Joint Infection	–

## Conclusion

In this study, a novel deep-learning-based model, termed SNF-NN, is introduced for predicting new drug-disease interactions using known drug-disease interactions, drug-related and disease-related similarity information. Three benchmark datasets are used to validate the performance of the proposed method. The accuracy and loss trends of SNF-NN are initially calculated to evaluate the training process of the proposed deep neural network model. Subsequently, the reliability and robustness of SNF-NN are verified by comparing its performance with nine baseline machine learning methods as well as seven state-of-the-art models for drug-disease interaction prediction. Although predicting novel RDIs is a very challenging problem due to the lack of known interactions, SNF-NN achieves remarkably higher evaluation metric values compared to the other methods with $AUC-ROC$ ranging from 0.879 to 0.931 and $AUC-PR$ from 0.856 to 0.903. In summary, the findings in this study indicate that heterogeneous similarity networks can be leveraged to develop superb computational drug repurposing methods that have both outstanding performance and improved interpretability. Such methods can significantly benefit from integrating similarity measures and deep learning models to predict novel drug-disease interactions.

## Supplementary information

**Additional file 1.** SND Benchmark Dataset.

## Data Availability

The data that support the findings of this study are available from a third party; in particular two benchmark datasets, namely Cdataset [17] and LRSSL [28] have been downloaded and used in this study.
