# Supplementary material for: SNF-NN: computational method to predict drug-disease interactions using similarity network fusion and neural networks
Source: BMC Bioinformatics. 2021 Jan 22;22:28. doi: 10.1186/s12859-020-03950-3 (PMC7821180; doi:10.1186/s12859-020-03950-3)
Supplement: Supplementary file 1 — Additional file 1. SND Benchmark Dataset. [file 12859_2020_3950_MOESM1_ESM.pdf]

SNF-NN: Computational Method To Predict Drug-Disease Interactions Using Similarity Network Fusion and Neural Networks

Supplementary Material

Tamer N. Jarada[*], Jon G. Rokne[*] and Reda Alhajj[*†■]

^*Department of Computer Science, University of Calgary, Calgary, Alberta, Canada

[†]Department of Computer Engineering, Istanbul Medipol University, Istanbul, Turkey

[■]Department of Health Informatics, University of Southern Denmark, Odense, Denmark

Contact author: Reda Alhajj, email: alhajj <at> ucalgary.ca

SND Benchmark Dataset

1 Drug-related similarity data

1.1 Drug Target Protein Interactions

Drugs tend to target human proteins via binding in order to enhance or inhibit biological functions performed by those proteins and, thus, affect the disease conditions and achieve desirable therapeutic effects.

Drug target protein interaction and bioactivity data were acquired from six widely used data sources. Drug-target interactions were retrieved from the DrugBank Database [1], Therapeutic Target Database (TTD) [2], and the Pharmacogenetics and Pharmacogenomics Knowledge Base (PharmGKB) [3]. Drug-target bioactivity data was retrieved from ChEMBL [4], the International Union of Basic and Clinical Pharmacology/British Pharmacological Society (IUPHAR/BPS) Guide to Pharmacology [5], and BindingDB [6]. The collected data was processed so that drug-target interactions were retained if the following criteria were satisfied:

- Human proteins are:

- Represented by unique UniProt Accession Number (AC) [7].
- Annotated as "reviewed" in the UniProtKB Database [8].

- Protein-ligand complexes have the following binding affinity values:
  - Inhibition Constant/Potency (K_i) affinity ≤ 10 $Micromolar$ (μM).
  - Dissociation Constant (K_d) affinity ≤ 10 (μM).
  - Median Effective Concentration (EC_{50}) affinity ≤ 10 (μM).
  - Median Inhibitory Concentration (IC_{50}) affinity ≤ 10 (μM).

A binary matrix was constructed to represent the drug-target protein interactions where each drug r is represented as a binary feature vector of UniProt target proteins, and the absence or presence of a drug-target relationship is denoted by 0 or 1, respectively.

Drugs with similar target proteins are more likely to have common biological functions and, therefore, share therapeutic indications. The similarity between drug pairs was calculated using the Jaccard coefficient and human target proteins.

$$S_{(jaccard)}(r_i, r_j) = \frac{|f(r_i) \cap f(r_j)|}{|f(r_i)|+|f(r_j)|-|f(r_i) \cap f(r_j)|}$$

where $|f(r_i)|$ and $|f(r_j)|$ are the total numbers of features in r_i and r_j, respectively. The intersection $|f(r_i) \cap f(r_j)|$ represents the number of common features between r_i and r_j. The $S_{(jaccard)}$ value is in the range [0, 1].

1.2 Drug Side Effects

Drug side effects or Adverse Drug Events (ADEs) resulting from off-targets present the ability of profiling human phenotypic traits related to drugs and eventually help to identify novel therapeutic uses for these drugs.

Drug side effects or clinically reported ADEs information was collected from SIDER Database [9], MetaADEDB [10], OFFSIDES [11], and CTD [12]. The retrieved data were annotated using DBANs, Medical Subject Headings (MeSH) [13], and Unified Medical Language System (UMLS) [14], which integrates many health and biomedical standards and vocabularies. Subsequently, the collected data was processed to exclude all duplicated drug-side-effect associations.

A binary matrix was constructed to represent the drug-side-effect associations where each drug r is represented as a binary feature vector of MeSH Unique ID of ADEs, and the absence or presence of a drug-ADE association is denoted by 0 or 1, respectively.

Drugs with similar ADEs are more probably to have common target proteins and therefore share similar underlying pathways. The similarity between drug pairs was calculated using the Jaccard coefficient and ADEs.

1.3 Drug Chemical Structures

Drug chemical structure information tends to point towards any transcriptional response similarity between drugs, and thus it is a powerful source of information for therapeutic repositioning opportunities.

Drug 2D chemical structure information was retrieved from PubChem [15]. Subsequently, CDK [16] was used to encode each drug into a binary feature vector of PubChem substructures.

A binary matrix was constructed to represent the drug-chemical-substructure associations where each drug r is represented as a binary feature vector of PubChem 2D chemical substructures, and the absence or presence of a drug-chemical-substructure association is denoted by 0 or 1, respectively.

Drugs with similar chemical structures are anticipated to have similar biological indications and, therefore, cure common diseases. The similarity between drug pairs was calculated using the Jaccard coefficient and the 2D chemical fingerprint descriptor.

1.4 Drug GO Annotation Targets

GO annotation describes the Molecular Functions (MF), Biological Processes (BP) and Cellular Components (CC) of genes and gene products and, therefore, provides a useful insight into the biology and functional interpretation of genes and gene products. Such insight significantly supports initiatives of identifying new therapeutic uses for existing drugs.

GO annotations of drug target-coding genes were obtained from the Gene Ontology Consortium Database [17]. Experimentally validated and literature-derived information (i.e., molecular functions, biological processes, cellular components) was utilized while computationally annotated information was kept out.

Binary matrices were constructed to represent the three drug pairwise semantic similarities where each drug r in the MF, BP, and CC semantic similarity matrices is represented as a binary feature vector the GO molecular function terms, GO biological process terms, and GO cellular component terms, respectively.

Drugs with semantic similar target-coding genes appear to share similar underlying mechanisms. The MF, BP, and CC semantic similarities between drug pairs were calculated using the information-based measure proposed in [18].

1.5 Drug Metabolism Enzymes

Drug metabolism refers to the set of biochemical processes and metabolic breakdown of drugs within any living organism. Drug metabolism is usually represented by the enzymatic alteration caused by the drug; hence, it can help identify similarities between drugs.

Drug metabolism enzymes information was retrieved from the Human Metabolome Database (HMDB) [19].

A binary matrix was constructed to represent the drug-metabolism-enzyme associations where each drug r is represented as a binary feature vector of enzymes, and the absence or presence of a drug-metabolism-enzyme association is denoted by 0 or 1, respectively.

Drugs with similar metabolism enzymes are more likely to share similar underlying pathways. The similarity between drug pairs was calculated using the Jaccard coefficient and metabolism enzymes.

1.6 Drug Protein Sequences

Drug protein sequence information refers to the amino acid sequence information of a protein, or part of a protein, which characterizes the protein post-translational modifications. Such information helps in studying the similarity between drugs and thus identifying novel drug repositioning candidates.

Drug target protein sequences were obtained from UniProt/SWISS-PROT KnowledgeBase [20] and KEGG [21].

A binary matrix was constructed to represent the drug-protein sequence associations, where each drug r is represented by a binary feature vector of protein sequences, and the absence or presence of a drug-protein-sequence association is denoted by 0 or 1, respectively.

Drugs with similar protein amino acid sequences tend to share common functional mechanisms. The similarity between drug pairs was calculated using the normalized average of the SW sequence alignment similarity scores of proteins [22].

$$S_{(proteins)}(r_a, r_b) = \frac{\sum_{i=1}^{|p(r_a)|} \sum_{j=1}^{|p(r_b)|} SW(p_i(r_a), p_j(r_b))}{|p(r_a)||p(r_b)|}$$

where $p(r_a)$ and $p(r_b)$ represent the target protein sets of drugs r_a and r_b, respectively; $|p(r_a)|$ and $|p(r_b)|$ are the total numbers of target proteins of drugs r_a and r_b, respectively, and $SW(p_i(r_a), p_j(r_b))$ is the sequence alignment similarity score between the two proteins $p_i(r_a)$ and $p_j(r_b)$. The $S_{(proteins)}$ value is in the range [0, 1].

1.7 Drug Anatomical Therapeutic Chemical Classification Codes

The Anatomical Therapeutic Chemical (ATC) classification system divides the active drug substances into different groups based on their organ or system on which they act as well as their therapeutic effect, pharmacological activities, and chemical characteristics. The ATC classification system consists of five different classification levels: (i) main anatomical or pharmacological groups, (ii) pharmacological or therapeutic subgroups, (iii) & (iv) chemical, pharmacological or therapeutic subgroups, and (v) chemical substances. Such a classification system provides useful insight into drugs and helps in predicting novel drug targets.

Drug-ATC classification codes were collected from the DrugBank Database [1].

A binary matrix was constructed to represent the drug-ATC classification code associations, where each drug r is represented by a binary feature vector of ATC classification codes, and the absence or presence of a drug-ATC association is denoted by 0 or 1, respectively.

Drugs with similar structures and classification codes appear to have similar medical indications. The similarity between drug pairs was calculated using the similarity measure proposed in [10].

1.8 Drug Pairwise Interactions

Drug-Drug Interactions (RRIs) refer to the adverse effects that may result from the concurrent consumption of two or more drugs. RRIs play a significant role in delaying, decreasing, or enhancing the absorption and expected molecular alterations of the concurrent consumed drugs.

Drug-drug interactions were collected from the DrugBank Database [1].

A binary adjacency matrix was constructed to represent the drug-drug interactions, where each drug r is represented by a binary feature vector of drugs, and the absence or presence of an RRI is denoted by 0 or 1, respectively.

Drugs with interactive chemicals are anticipated to have similar characteristics and biological functions.

2 Disease-related similarity data

2.1 Disease Genes

Disease-Gene Associations (DGAs) describe the relationships between genetic diseases and the genes associated with them. Such associations help in understanding the underlying mechanism of complex diseases, and therefore they support the efforts of developing preventive and therapeutic solutions.

Curated disease-gene associations were collected from UniProt/SWISS-PROT KnowledgeBase [20], the Comparative Toxicogenomics Database (CTD) [23], and the Clinical Genome Resource (ClinGen)

[24]. Genes were mapped to their UniProt accession numbers [7].

Human Phenotype Ontology (HPO) disease-gene associations were also retrieved from the HPO KnowledgeBase [25]. Diseases were mapped from their Disease Ontology Identifiers (DOIDs) to their UMLS's concept unique identifiers.

Literature-based disease-gene associations were extracted from Medline abstracts published in the period (January 1970-December 2019) using the BeFree text mining tool [26]. BeFree consists of two modules, namely Biomedical Named Entity Recognition (BioNER) and Relation Extraction (RE). BioNER utilizes gene and disease dictionaries to apply fuzzy and pattern matching methods in order to recognize gene and disease entities mentioned in the literature [27]. Subsequently, RE identifies DGAs using the exploitation of semantic and morphosyntactic information from the text extracted from the literature [28]. Genes were mapped to their UniProt accession numbers [7].

Binary matrices were constructed to represent the curated, HPO, and literature-based DGAs where each disease d is represented as a binary feature vector of genes, and the absence or presence of a DGA is denoted by 0 or 1, respectively.

Diseases associated with similar genes tend to share similar underlying mechanisms. The similarity between disease pairs was calculated using the Jaccard coefficient and curated, HOP, and literature-based genes, separately.

2.2 Disease Variants

Disease-Variant Associations (DVAs) relate to the relationships between genetic diseases and the genetic variants associated with them. Variants refer to the gene's DNA sequence changes that are caused by a disease. Gene variants can alter the gene functionality (e.g., produce a non-functioning protein) and, thus, provide useful insight into disease conditions.

Curated disease-variant associations were obtained from UniProt/SWISS-PROT KnowledgeBase [20] and the GWASdb Database of human genetic variants identified by genome-wide association studies [29]. Variants were mapped to their Single Nucleotide Polymorphisms (SNPs) using the NCBI Database of Genetic Variation (dbSNP) [30].

Literature-based disease-variant associations were extracted from Medline abstracts published in the period (January 1970-December 2019) using both the SETH text mining tool [31] and the BeFree tool. SETH extracts gene variants and maps them to their dbSNP's SNPs, while the RE module of the BeFree tool identifies DVAs from the extracted information.

Binary matrices were constructed to represent the curated and literature-based DVAs where each disease d is represented as a binary feature vector of variants, and the absence or presence of a DVA is denoted by 0 or 1, respectively.

Diseases associated with similar variants are expected to share similar underlying pathways. The similarity between disease pairs was calculated using the Jaccard coefficient and curated and literature-based variants, separately.

2.3 Disease MicroRNAs

Disease-MicroRNA Associations (DMAs) refer to the single-stranded non-coding RNA sequences associated with a disease. MicroRNAs (miRNAs) regulate up to 60% of protein-encoding genes. They are also considered as key mediators of the host response to diseases [32].

Disease-microRNA associations were collected from the DincRNA Database [33]. Diseases were mapped from their DOIDs to their UMLS's CUIs.

A binary matrix was constructed to represent the disease-miRNA associations, where each disease d is represented by a binary feature vector of miRNAs, and the absence or presence of a DMA is denoted by 0 or 1, respectively.

Diseases that affect similar miRNAs are expected to have similar underlying pathways. The similarity between disease pairs was calculated using the Jaccard coefficient and miRNAs.

2.4 Disease Long Non-coding RNAs

Disease-Long Non-coding RNAs Associations (DLAs) describe the relationship between the genetic diseases and the Long Non-coding RNAs (lncRNAs) associated with them. lncRNAs are RNA transcripts with lengths exceeding 200 nucleotides. Despite lacking protein-coding potential, lncRNAs are emerging as important regulators in gene expression networks for their roles in modulating mRNA stability and controlling nuclear architecture and transcription as well as translation and post-translational modifications in the cytoplasm [34].

Disease-lncRNAs associations were collected from the DincRNA Database [33]. Diseases were mapped from their DOIDs to their UMLS's CUIs.

A binary matrix was constructed to represent the disease-lncRNA associations, where each disease d is represented by a binary feature vector of lncRNAs, and the absence or presence of a DLA is denoted by 0 or 1, respectively.

Diseases with similar lncRNAs tend to share similar cellular functional effects. The similarity between disease pairs was calculated using the Jaccard coefficient and lncRNAs.

2.5 Disease HPO Phenotypes

Disease phenotypes present the observable characteristics of the disease and tend to describe the quality of an organism (e.g., morphology, development, behaviour) as opposed to the organism's genotype [35].

Disease phenotypes associations were retrieved from the HPO KnowledgeBase [25]. Diseases were mapped from their DOIDs to their UMLS's CUIs.

A binary matrix was constructed to represent the disease-phenotype associations, where each disease d is represented by a binary feature vector of phenotypes, and the absence or presence of a phenotype is denoted by 0 or 1, respectively.

Diseases can be defined using their phenotypes; therefore, diseases with similar phenotypes are anticipated to share similar underlying pathways. The similarity between disease pairs was calculated using the Jaccard coefficient and phenotypes.

2.6 Inferred Disease-Disease Associations

Inferred disease pairwise similarity methods raise great attention lately for their key role in identifying disease-causing genes [36] and predicting novel drug indications [37]. Such computational methods quantify the shared characteristics of disease pairs based on semantic and/or functional information. Semantic-based methods use terms of gene ontology [38] and human phenotype ontology [25] to calculate pairwise disease similarity, while function-based methods use functional associations of disease genetic entities (e.g., genes, miRNAs, lncRNAs) [39].

Inferred Disease-Disease Associations (IDDAs) based on six different computational methods, namely Resnik, Lin, Wang, PSB, SemFunSim, and DisGeNET, were collected from the DincRNA Database [33] and the DisGeNET KnowledgeBase [40]. Diseases in all IDDAs datasets were mapped from their DOIDs to their UMLS's CUIs.

Resnik [41] introduced a semantic similarity measure in an IS-A taxonomy based on the notion of information content. However, Lin [42] used information-theoretic definition to compute semantic pairwise similarity scores. Moreover, Wang [43] developed a novel semantic similarity measure based on Go terms. Furthermore, Mathur [44] leveraged implicit semantic similarity to propose the PSB similarity measure. In addition, Cheng [45] integrated semantic and gene functional information to present the SemFunSim similarity measure. Finally, Piñero [40] utilized curated disease genes and variants to compute disease pairwise similarity reported in the DisGeNET KnowledgeBase.

Binary adjacency matrices were constructed to represent the disease-disease associations, where each disease d is represented by a binary feature vector of diseases, and the absence or presence of an IDDA is denoted by 0 or 1, respectively.

Diseases with high similarity scores are anticipated to have similar characteristics, genomic effects, and underlying pathways.

Table 1 summarizes the performance metrics of all trained deep neural network models with their hyperparameters settings

Number of hidden layers	Number of neurons	Dropout rate	Activation function	AUC	AUC-PR	F-score	Prec	Rec	Spec	Acc	MCC
4	300	0.35	ReLU	0.790	0.800	0.726	0.725	0.728	0.724	0.726	0.452
3	200	0.4	ReLU	0.786	0.790	0.732	0.714	0.750	0.700	0.725	0.451
2	100	0.4	ReLU	0.786	0.791	0.725	0.732	0.719	0.737	0.728	0.456
3	100	0.4	ReLU	0.785	0.793	0.730	0.733	0.728	0.735	0.731	0.462
1	100	0.4	ReLU	0.785	0.789	0.716	0.716	0.716	0.717	0.716	0.433
1	300	0.3	ReLU	0.784	0.791	0.726	0.731	0.721	0.735	0.728	0.455
1	500	0.3	ReLU	0.783	0.791	0.730	0.716	0.745	0.704	0.724	0.449
1	400	0.45	ReLU	0.782	0.786	0.726	0.727	0.724	0.728	0.726	0.452
2	200	0.3	ReLU	0.782	0.787	0.724	0.699	0.750	0.678	0.714	0.428
1	500	0.5	ReLU	0.782	0.790	0.723	0.733	0.713	0.739	0.726	0.453
1	300	0.5	ReLU	0.781	0.787	0.721	0.739	0.704	0.751	0.728	0.456
2	200	0.4	ReLU	0.781	0.787	0.718	0.716	0.720	0.715	0.718	0.435
4	500	0.35	ReLU	0.781	0.791	0.715	0.722	0.709	0.727	0.718	0.436
1	400	0.3	ReLU	0.781	0.789	0.725	0.729	0.721	0.732	0.727	0.453
1	300	0.4	ReLU	0.781	0.780	0.720	0.731	0.708	0.740	0.724	0.448
2	300	0.5	ReLU	0.780	0.787	0.709	0.744	0.678	0.767	0.722	0.446
3	400	0.3	ReLU	0.780	0.787	0.728	0.710	0.747	0.696	0.721	0.443
1	200	0.5	ReLU	0.780	0.785	0.726	0.731	0.721	0.735	0.728	0.455
2	500	0.4	ReLU	0.780	0.784	0.729	0.713	0.746	0.699	0.723	0.446
5	500	0.5	ReLU	0.780	0.785	0.724	0.722	0.727	0.720	0.723	0.446
2	300	0.4	ReLU	0.780	0.782	0.720	0.718	0.721	0.717	0.719	0.439
5	200	0.35	ReLU	0.780	0.781	0.723	0.717	0.729	0.711	0.720	0.441
5	100	0.4	ReLU	0.780	0.794	0.728	0.714	0.742	0.703	0.723	0.446
3	300	0.4	ReLU	0.779	0.785	0.727	0.679	0.783	0.630	0.707	0.418
4	200	0.3	ReLU	0.779	0.796	0.729	0.701	0.758	0.677	0.718	0.437
1	100	0.35	ReLU	0.779	0.783	0.716	0.716	0.716	0.716	0.716	0.432
3	100	0.5	ReLU	0.779	0.792	0.719	0.693	0.747	0.669	0.708	0.417
1	500	0.4	ReLU	0.779	0.783	0.724	0.704	0.744	0.687	0.716	0.433
3	200	0.45	ReLU	0.779	0.782	0.716	0.706	0.727	0.697	0.712	0.424
5	400	0.35	ReLU	0.779	0.784	0.727	0.714	0.739	0.704	0.722	0.444
4	300	0.35	ReLU	0.779	0.787	0.723	0.730	0.717	0.735	0.726	0.452
2	300	0.35	ReLU	0.779	0.780	0.725	0.720	0.730	0.716	0.723	0.446
1	500	0.45	ReLU	0.779	0.784	0.719	0.707	0.731	0.696	0.714	0.428
3	300	0.3	ReLU	0.778	0.784	0.724	0.703	0.746	0.685	0.715	0.431
4	200	0.35	ReLU	0.778	0.781	0.720	0.730	0.710	0.737	0.724	0.447
1	300	0.45	ReLU	0.778	0.782	0.716	0.716	0.716	0.716	0.716	0.432
2	400	0.5	ReLU	0.778	0.781	0.719	0.700	0.739	0.683	0.711	0.423
3	300	0.35	ReLU	0.778	0.778	0.721	0.706	0.737	0.693	0.715	0.431
1	400	0.35	ReLU	0.778	0.785	0.719	0.704	0.734	0.691	0.713	0.426

Continued on next page

Table 1 – continued from previous page

Number of hidden layers	Number of neurons	Dropout rate	Activation function	AUC	AUC-PR	F-score	Prec	Rec	Spec	Acc	MCC
1	100	0.5	ReLU	0.778	0.783	0.713	0.727	0.698	0.738	0.718	0.437
4	200	0.45	ReLU	0.778	0.783	0.718	0.707	0.730	0.697	0.713	0.427
2	400	0.45	ReLU	0.778	0.787	0.714	0.713	0.714	0.713	0.714	0.427
2	500	0.3	ReLU	0.777	0.780	0.719	0.706	0.733	0.694	0.714	0.428
2	400	0.3	ReLU	0.777	0.778	0.726	0.689	0.767	0.654	0.710	0.424
3	200	0.3	ReLU	0.777	0.783	0.717	0.707	0.727	0.698	0.713	0.425
3	100	0.45	ReLU	0.777	0.785	0.714	0.710	0.718	0.707	0.713	0.426
1	200	0.35	ReLU	0.777	0.780	0.719	0.682	0.759	0.647	0.703	0.408
4	200	0.4	ReLU	0.777	0.785	0.722	0.708	0.737	0.696	0.716	0.433
1	100	0.45	ReLU	0.777	0.777	0.713	0.711	0.715	0.709	0.712	0.424
5	300	0.45	ReLU	0.777	0.779	0.723	0.682	0.768	0.642	0.705	0.414
3	500	0.35	ReLU	0.777	0.781	0.719	0.707	0.732	0.697	0.714	0.429
4	500	0.45	ReLU	0.777	0.781	0.719	0.725	0.713	0.729	0.721	0.442
1	100	0.4	ReLU	0.777	0.779	0.711	0.709	0.713	0.708	0.711	0.421
3	400	0.5	ReLU	0.777	0.779	0.716	0.731	0.702	0.742	0.722	0.443
5	400	0.5	ReLU	0.777	0.782	0.722	0.713	0.732	0.705	0.718	0.437
3	200	0.5	ReLU	0.777	0.784	0.709	0.737	0.684	0.756	0.720	0.441
1	400	0.5	ReLU	0.777	0.779	0.719	0.713	0.725	0.708	0.717	0.434
2	400	0.4	ReLU	0.777	0.780	0.717	0.715	0.719	0.714	0.716	0.433
5	300	0.5	ReLU	0.777	0.786	0.720	0.676	0.771	0.630	0.701	0.405
3	400	0.4	ReLU	0.777	0.784	0.726	0.699	0.755	0.675	0.715	0.431
4	400	0.35	ReLU	0.777	0.781	0.721	0.711	0.730	0.704	0.717	0.434
1	200	0.3	ReLU	0.776	0.779	0.714	0.705	0.723	0.697	0.710	0.420
1	200	0.45	ReLU	0.776	0.783	0.711	0.720	0.702	0.727	0.715	0.429
1	500	0.35	ReLU	0.776	0.781	0.718	0.707	0.729	0.697	0.713	0.427
3	300	0.5	ReLU	0.776	0.784	0.717	0.728	0.706	0.736	0.721	0.442
3	100	0.35	ReLU	0.776	0.777	0.723	0.707	0.740	0.693	0.716	0.433
2	200	0.5	ReLU	0.776	0.783	0.708	0.725	0.692	0.737	0.714	0.429
2	100	0.5	ReLU	0.775	0.781	0.717	0.718	0.716	0.719	0.718	0.435
3	100	0.3	ReLU	0.775	0.779	0.719	0.708	0.730	0.699	0.715	0.429
4	200	0.5	ReLU	0.775	0.786	0.712	0.717	0.707	0.721	0.714	0.428
2	500	0.45	ReLU	0.775	0.780	0.714	0.700	0.728	0.688	0.708	0.416
2	200	0.45	ReLU	0.775	0.780	0.714	0.719	0.710	0.723	0.716	0.433
3	400	0.45	ReLU	0.775	0.780	0.719	0.717	0.722	0.715	0.718	0.437
2	300	0.45	ReLU	0.775	0.778	0.714	0.699	0.729	0.687	0.708	0.416
3	300	0.45	ReLU	0.775	0.780	0.717	0.693	0.743	0.671	0.707	0.416
2	200	0.35	ReLU	0.775	0.779	0.713	0.693	0.735	0.675	0.705	0.410
2	300	0.3	ReLU	0.775	0.774	0.720	0.689	0.754	0.659	0.707	0.415
4	500	0.5	ReLU	0.775	0.774	0.708	0.729	0.689	0.743	0.716	0.433
2	400	0.35	ReLU	0.775	0.775	0.720	0.725	0.715	0.728	0.721	0.443
2	500	0.5	ReLU	0.775	0.777	0.712	0.734	0.692	0.749	0.720	0.441

Continued on next page

Table 1 – continued from previous page

Number of hidden layers	Number of neurons	Dropout rate	Activation function	AUC	AUC-PR	F-score	Prec	Rec	Spec	Acc	MCC
5	500	0.45	ReLU	0.774	0.782	0.718	0.711	0.725	0.705	0.715	0.430
3	500	0.45	ReLU	0.774	0.775	0.706	0.717	0.696	0.725	0.711	0.421
4	100	0.35	ReLU	0.774	0.778	0.711	0.688	0.735	0.667	0.701	0.403
4	300	0.5	ReLU	0.774	0.779	0.718	0.711	0.727	0.704	0.715	0.431
4	400	0.5	ReLU	0.774	0.778	0.714	0.717	0.711	0.719	0.715	0.430
4	100	0.3	ReLU	0.774	0.782	0.710	0.710	0.711	0.710	0.710	0.421
2	500	0.35	ReLU	0.774	0.784	0.719	0.702	0.736	0.688	0.712	0.424
1	200	0.4	ReLU	0.774	0.776	0.713	0.700	0.727	0.688	0.707	0.415
5	100	0.35	ReLU	0.773	0.773	0.719	0.683	0.759	0.648	0.703	0.409
5	400	0.45	ReLU	0.773	0.777	0.722	0.709	0.736	0.698	0.717	0.434
3	500	0.3	ReLU	0.773	0.780	0.727	0.704	0.752	0.684	0.718	0.437
4	300	0.45	ReLU	0.773	0.776	0.714	0.703	0.726	0.694	0.710	0.420
4	400	0.45	ReLU	0.773	0.778	0.709	0.693	0.726	0.679	0.702	0.405
1	400	0.4	ReLU	0.773	0.777	0.713	0.696	0.731	0.680	0.706	0.412
3	500	0.5	ReLU	0.773	0.774	0.711	0.733	0.691	0.748	0.719	0.440
2	100	0.35	ReLU	0.773	0.776	0.717	0.704	0.730	0.693	0.712	0.424
3	400	0.35	ReLU	0.773	0.779	0.725	0.720	0.729	0.717	0.723	0.446
5	300	0.35	ReLU	0.772	0.773	0.721	0.715	0.727	0.710	0.718	0.437
5	500	0.35	ReLU	0.772	0.780	0.719	0.683	0.759	0.648	0.704	0.410
4	300	0.4	ReLU	0.772	0.788	0.713	0.721	0.706	0.728	0.717	0.433
1	100	0.3	ReLU	0.772	0.777	0.713	0.691	0.735	0.672	0.703	0.408
3	200	0.35	ReLU	0.772	0.773	0.715	0.699	0.731	0.685	0.708	0.416
2	100	0.45	ReLU	0.772	0.777	0.708	0.708	0.708	0.708	0.708	0.416
2	100	0.3	ReLU	0.771	0.772	0.715	0.706	0.724	0.698	0.711	0.422
3	500	0.4	ReLU	0.771	0.781	0.725	0.706	0.744	0.690	0.717	0.435
5	200	0.45	ReLU	0.768	0.771	0.714	0.658	0.780	0.595	0.688	0.382
4	300	0.3	ReLU	0.768	0.780	0.720	0.713	0.728	0.706	0.717	0.434
4	100	0.45	ReLU	0.767	0.773	0.710	0.651	0.781	0.581	0.681	0.369
5	100	0.3	ReLU	0.767	0.783	0.713	0.689	0.739	0.666	0.702	0.406
4	100	0.45	TanH	0.763	0.774	0.708	0.694	0.722	0.682	0.702	0.404
5	100	0.4	TanH	0.762	0.767	0.705	0.702	0.708	0.699	0.704	0.407
5	200	0.3	ReLU	0.759	0.784	0.716	0.698	0.735	0.682	0.708	0.417
4	100	0.35	TanH	0.759	0.769	0.704	0.704	0.704	0.704	0.704	0.409
4	100	0.3	TanH	0.759	0.764	0.704	0.706	0.701	0.709	0.705	0.409
5	100	0.35	TanH	0.759	0.771	0.710	0.700	0.721	0.691	0.706	0.412
5	100	0.45	ReLU	0.759	0.738	0.714	0.615	0.850	0.468	0.659	0.345
3	100	0.35	TanH	0.759	0.763	0.707	0.695	0.718	0.685	0.702	0.404
5	300	0.5	TanH	0.758	0.762	0.705	0.685	0.727	0.665	0.696	0.393
3	100	0.4	TanH	0.758	0.765	0.702	0.694	0.710	0.687	0.698	0.397
5	200	0.4	TanH	0.757	0.760	0.702	0.693	0.711	0.685	0.698	0.396
4	400	0.4	ReLU	0.757	0.780	0.720	0.691	0.750	0.665	0.708	0.417

Continued on next page

Table 1 – continued from previous page

Number of hidden layers	Number of neurons	Dropout rate	Activation function	AUC	AUC-PR	F-score	Prec	Rec	Spec	Acc	MCC
5	100	0.45	TanH	0.757	0.767	0.694	0.696	0.692	0.698	0.695	0.390
5	200	0.45	TanH	0.757	0.771	0.708	0.700	0.715	0.694	0.705	0.409
4	200	0.35	TanH	0.757	0.764	0.708	0.698	0.719	0.688	0.704	0.407
5	200	0.5	ReLU	0.756	0.744	0.707	0.622	0.818	0.503	0.661	0.339
2	100	0.35	TanH	0.756	0.755	0.707	0.701	0.714	0.695	0.704	0.409
4	200	0.45	TanH	0.756	0.756	0.708	0.698	0.719	0.689	0.704	0.408
1	100	0.45	TanH	0.756	0.759	0.694	0.702	0.687	0.708	0.697	0.395
2	100	0.45	TanH	0.756	0.757	0.705	0.695	0.716	0.685	0.701	0.401
4	100	0.4	TanH	0.756	0.768	0.704	0.697	0.711	0.690	0.701	0.402
3	100	0.45	TanH	0.756	0.763	0.704	0.699	0.710	0.694	0.702	0.403
4	200	0.4	TanH	0.755	0.760	0.703	0.690	0.716	0.678	0.697	0.394
4	100	0.5	TanH	0.755	0.761	0.699	0.682	0.716	0.667	0.691	0.383
1	100	0.5	TanH	0.755	0.759	0.702	0.687	0.718	0.673	0.696	0.392
3	200	0.5	TanH	0.755	0.762	0.697	0.678	0.717	0.659	0.688	0.377
5	500	0.5	TanH	0.754	0.756	0.707	0.693	0.721	0.680	0.701	0.402
1	200	0.3	TanH	0.754	0.749	0.705	0.700	0.709	0.696	0.703	0.405
5	200	0.35	TanH	0.754	0.759	0.709	0.696	0.723	0.684	0.703	0.406
5	200	0.4	ReLU	0.754	0.780	0.705	0.729	0.683	0.746	0.715	0.430
1	100	0.35	TanH	0.754	0.754	0.706	0.699	0.712	0.693	0.703	0.406
4	400	0.3	ReLU	0.754	0.773	0.713	0.695	0.731	0.680	0.705	0.411
2	100	0.4	TanH	0.754	0.756	0.706	0.696	0.717	0.687	0.702	0.404
2	200	0.45	TanH	0.754	0.754	0.701	0.695	0.706	0.691	0.699	0.397
1	100	0.4	TanH	0.754	0.754	0.698	0.692	0.704	0.687	0.696	0.391
3	100	0.5	TanH	0.754	0.761	0.699	0.678	0.721	0.657	0.689	0.379
1	500	0.35	TanH	0.753	0.750	0.708	0.696	0.721	0.685	0.703	0.406
1	200	0.45	TanH	0.753	0.744	0.704	0.694	0.714	0.685	0.699	0.399
4	100	0.5	ReLU	0.753	0.738	0.702	0.579	0.892	0.351	0.622	0.289
1	400	0.3	TanH	0.753	0.747	0.700	0.711	0.690	0.719	0.705	0.409
5	200	0.3	TanH	0.753	0.746	0.708	0.699	0.716	0.691	0.704	0.408
4	200	0.5	TanH	0.753	0.752	0.701	0.675	0.729	0.649	0.689	0.379
4	300	0.35	TanH	0.753	0.744	0.705	0.688	0.723	0.672	0.697	0.395
1	500	0.3	TanH	0.753	0.748	0.705	0.704	0.705	0.704	0.705	0.409
3	300	0.5	TanH	0.752	0.757	0.704	0.685	0.723	0.668	0.696	0.392
2	100	0.3	TanH	0.752	0.754	0.704	0.701	0.706	0.699	0.703	0.405
2	300	0.35	TanH	0.752	0.750	0.710	0.704	0.716	0.699	0.708	0.415
2	100	0.5	TanH	0.752	0.760	0.694	0.672	0.717	0.649	0.683	0.367
5	500	0.45	TanH	0.752	0.744	0.708	0.699	0.717	0.691	0.704	0.408
1	100	0.3	TanH	0.752	0.747	0.701	0.694	0.709	0.687	0.698	0.396
1	300	0.35	TanH	0.752	0.747	0.698	0.701	0.695	0.704	0.699	0.398
5	100	0.5	TanH	0.752	0.760	0.699	0.678	0.722	0.657	0.689	0.379
1	500	0.4	TanH	0.752	0.748	0.706	0.697	0.716	0.689	0.702	0.405

Continued on next page

Table 1 – continued from previous page

Number of hidden layers	Number of neurons	Dropout rate	Activation function	AUC	AUC-PR	F-score	Prec	Rec	Spec	Acc	MCC
2	300	0.45	TanH	0.752	0.746	0.707	0.698	0.717	0.689	0.703	0.406
3	300	0.45	TanH	0.752	0.749	0.698	0.702	0.694	0.706	0.700	0.400
5	200	0.5	TanH	0.751	0.756	0.697	0.678	0.716	0.660	0.688	0.377
3	200	0.45	TanH	0.751	0.754	0.701	0.704	0.698	0.706	0.702	0.404
1	400	0.35	TanH	0.751	0.745	0.704	0.697	0.711	0.691	0.701	0.403
1	200	0.35	TanH	0.751	0.747	0.696	0.702	0.689	0.708	0.699	0.397
5	300	0.45	TanH	0.751	0.752	0.699	0.683	0.716	0.668	0.692	0.384
1	300	0.3	TanH	0.751	0.744	0.701	0.702	0.701	0.702	0.701	0.403
2	300	0.5	TanH	0.751	0.751	0.704	0.696	0.711	0.690	0.700	0.401
2	200	0.35	TanH	0.751	0.748	0.697	0.700	0.695	0.702	0.699	0.397
1	400	0.4	TanH	0.751	0.747	0.691	0.705	0.677	0.716	0.697	0.393
5	400	0.35	TanH	0.750	0.745	0.709	0.701	0.718	0.693	0.706	0.411
1	500	0.5	TanH	0.750	0.745	0.701	0.700	0.703	0.698	0.701	0.401
2	200	0.5	TanH	0.750	0.753	0.697	0.674	0.722	0.651	0.687	0.374
1	300	0.5	TanH	0.750	0.748	0.697	0.682	0.713	0.668	0.691	0.382
2	300	0.4	TanH	0.750	0.749	0.705	0.703	0.706	0.701	0.704	0.408
4	300	0.45	TanH	0.750	0.757	0.706	0.692	0.720	0.680	0.700	0.400
1	400	0.45	TanH	0.750	0.745	0.702	0.693	0.711	0.684	0.698	0.396
2	200	0.4	TanH	0.750	0.748	0.702	0.695	0.709	0.688	0.699	0.397
3	300	0.4	TanH	0.750	0.746	0.704	0.690	0.717	0.678	0.698	0.396
2	200	0.3	TanH	0.749	0.746	0.696	0.706	0.687	0.713	0.700	0.400
5	100	0.3	TanH	0.749	0.754	0.701	0.689	0.713	0.679	0.696	0.392
3	500	0.5	TanH	0.749	0.743	0.709	0.689	0.730	0.671	0.700	0.402
3	100	0.3	TanH	0.748	0.750	0.704	0.696	0.712	0.688	0.700	0.401
2	400	0.5	TanH	0.748	0.749	0.696	0.678	0.714	0.661	0.688	0.376
2	400	0.35	TanH	0.748	0.739	0.707	0.701	0.714	0.696	0.705	0.410
4	500	0.5	TanH	0.748	0.746	0.702	0.669	0.738	0.636	0.687	0.375
1	200	0.4	TanH	0.748	0.742	0.696	0.703	0.690	0.708	0.699	0.398
5	300	0.4	TanH	0.748	0.745	0.706	0.699	0.714	0.692	0.703	0.406
1	300	0.4	TanH	0.748	0.743	0.693	0.703	0.684	0.711	0.697	0.394
1	400	0.5	TanH	0.748	0.747	0.699	0.680	0.719	0.662	0.691	0.382
1	200	0.5	TanH	0.748	0.748	0.695	0.682	0.709	0.669	0.689	0.378
3	200	0.4	TanH	0.747	0.745	0.697	0.693	0.701	0.689	0.695	0.391
5	300	0.35	TanH	0.747	0.739	0.705	0.696	0.713	0.689	0.701	0.402
2	500	0.4	TanH	0.747	0.738	0.705	0.698	0.712	0.692	0.702	0.404
1	500	0.45	TanH	0.747	0.743	0.695	0.701	0.690	0.705	0.697	0.395
5	400	0.4	TanH	0.747	0.745	0.696	0.704	0.689	0.710	0.699	0.399
4	500	0.45	TanH	0.747	0.731	0.714	0.692	0.736	0.672	0.704	0.409
5	400	0.45	TanH	0.747	0.739	0.710	0.695	0.724	0.682	0.703	0.407
1	300	0.45	TanH	0.747	0.743	0.692	0.699	0.684	0.706	0.695	0.390
4	400	0.5	TanH	0.747	0.747	0.704	0.682	0.728	0.660	0.694	0.389

Continued on next page

Table 1 – continued from previous page

Number of hidden layers	Number of neurons	Dropout rate	Activation function	AUC	AUC-PR	F-score	Prec	Rec	Spec	Acc	MCC
4	300	0.4	TanH	0.747	0.747	0.705	0.688	0.723	0.672	0.698	0.396
3	200	0.3	TanH	0.747	0.742	0.707	0.696	0.718	0.686	0.702	0.404
2	500	0.5	TanH	0.747	0.743	0.700	0.681	0.720	0.662	0.691	0.384
5	300	0.3	ReLU	0.747	0.778	0.702	0.709	0.695	0.714	0.705	0.409
2	400	0.4	TanH	0.747	0.738	0.700	0.705	0.696	0.708	0.702	0.404
2	400	0.45	TanH	0.746	0.738	0.709	0.705	0.713	0.701	0.707	0.414
5	400	0.5	TanH	0.746	0.738	0.700	0.676	0.725	0.653	0.689	0.379
2	500	0.45	TanH	0.746	0.746	0.690	0.696	0.684	0.702	0.693	0.386
4	300	0.5	TanH	0.745	0.748	0.700	0.675	0.727	0.650	0.688	0.378
4	200	0.3	TanH	0.745	0.739	0.696	0.699	0.693	0.702	0.698	0.395
3	400	0.4	TanH	0.745	0.737	0.704	0.699	0.708	0.695	0.702	0.403
3	300	0.35	TanH	0.745	0.744	0.700	0.707	0.693	0.712	0.703	0.405
4	400	0.3	TanH	0.745	0.733	0.710	0.690	0.732	0.671	0.701	0.403
3	400	0.45	TanH	0.744	0.742	0.706	0.695	0.718	0.684	0.701	0.403
4	400	0.45	TanH	0.744	0.740	0.707	0.694	0.720	0.682	0.701	0.402
5	100	0.5	ReLU	0.744	0.720	0.701	0.592	0.859	0.407	0.633	0.298
4	500	0.3	TanH	0.744	0.733	0.709	0.695	0.723	0.682	0.703	0.406
5	300	0.3	TanH	0.744	0.739	0.707	0.696	0.717	0.687	0.702	0.405
3	200	0.35	TanH	0.744	0.742	0.692	0.697	0.688	0.701	0.694	0.389
2	500	0.35	TanH	0.743	0.734	0.706	0.692	0.720	0.680	0.700	0.400
3	400	0.5	TanH	0.743	0.743	0.699	0.676	0.722	0.654	0.688	0.377
4	400	0.35	TanH	0.743	0.739	0.703	0.697	0.709	0.692	0.700	0.401
2	400	0.3	TanH	0.743	0.735	0.698	0.709	0.687	0.718	0.703	0.406
4	400	0.4	TanH	0.743	0.732	0.710	0.704	0.715	0.700	0.707	0.415
3	500	0.4	TanH	0.743	0.740	0.704	0.696	0.712	0.689	0.700	0.401
5	500	0.35	TanH	0.743	0.724	0.707	0.706	0.709	0.705	0.707	0.414
4	500	0.4	TanH	0.742	0.731	0.707	0.694	0.719	0.684	0.701	0.403
3	500	0.3	TanH	0.742	0.728	0.704	0.698	0.710	0.693	0.701	0.402
3	400	0.35	TanH	0.742	0.734	0.699	0.708	0.690	0.716	0.703	0.406
2	500	0.3	TanH	0.742	0.729	0.704	0.689	0.720	0.674	0.697	0.395
5	500	0.3	TanH	0.741	0.727	0.705	0.698	0.711	0.692	0.702	0.403
3	500	0.35	TanH	0.740	0.730	0.706	0.694	0.718	0.684	0.701	0.401
3	300	0.3	TanH	0.740	0.728	0.702	0.695	0.710	0.688	0.699	0.398
4	500	0.35	TanH	0.739	0.732	0.701	0.692	0.711	0.684	0.697	0.395
4	300	0.3	TanH	0.739	0.726	0.691	0.703	0.679	0.713	0.696	0.392
3	500	0.45	TanH	0.739	0.741	0.691	0.704	0.679	0.715	0.697	0.394
2	300	0.3	TanH	0.738	0.727	0.697	0.703	0.691	0.707	0.699	0.398
5	400	0.3	TanH	0.736	0.721	0.709	0.700	0.717	0.692	0.705	0.410
4	500	0.4	ReLU	0.736	0.764	0.703	0.696	0.709	0.690	0.700	0.399
3	400	0.3	TanH	0.736	0.719	0.705	0.697	0.713	0.690	0.702	0.403
5	500	0.4	TanH	0.735	0.713	0.690	0.706	0.674	0.719	0.696	0.393

Continued on next page

Table 1 – continued from previous page

Number of hidden layers	Number of neurons	Dropout rate	Activation function	AUC	AUC-PR	F-score	Prec	Rec	Spec	Acc	MCC
4	500	0.3	ReLU	0.729	0.774	0.708	0.683	0.734	0.660	0.697	0.395
5	400	0.3	ReLU	0.725	0.776	0.698	0.708	0.689	0.716	0.702	0.404
5	300	0.4	ReLU	0.724	0.781	0.706	0.693	0.720	0.681	0.700	0.401
5	500	0.4	ReLU	0.704	0.767	0.648	0.764	0.563	0.826	0.694	0.403
5	400	0.4	ReLU	0.697	0.770	0.696	0.685	0.706	0.676	0.691	0.382
5	500	0.3	ReLU	0.696	0.771	0.696	0.688	0.704	0.681	0.692	0.385

Table 1: Nested cross-validation results of the trained deep neural network models with their hyperparameters settings

Table 2 summarizes the SNF-NN predicted drug-disease interactions

Drug name	DrugBank ID	Disease name	Disease CUI
Etanercept	DB00005	Heartburn	C0018834
Peginterferon alfa-2a	DB00008	Enterobacter Pneumonia	C1096258
Goserelin	DB00014	Allergic Rhinitis (Disorder)	C2607914
Salmon Calcitonin	DB00017	Salmonella Sepsis	C0152486
Gramicidin D	DB00027	Nosocomial Pneumonia	C0949083
Gramicidin D	DB00027	Syphilis	C0039128
Gramicidin D	DB00027	Follicular Thyroid Carcinoma	C0206682
Gramicidin D	DB00027	Blastomycosis	C0005716
Gramicidin D	DB00027	Infective Otitis Media	C0729586
Insulin Human	DB00030	Allergic Conjunctivitis	C0009766
Desmopressin	DB00035	Otitis Externa	C0029878
Coagulation factor VIIa Recombinant Human	DB00036	Vitamin B 12 Deficiency	C0042847
Omalizumab	DB00043	Typhus, Epidemic Louse-Borne	C0041473
Somatropin recombinant	DB00052	Schizophrenia	C0036341
Somatropin recombinant	DB00052	Chancroids	C0007947
Somatropin recombinant	DB00052	Atrophy of Vulva	C0156393
Trastuzumab	DB00072	Tetanus	C0039614
Daclizumab	DB00111	Leukemia, Myelocytic, Acute	C0023467
Xanthophyll	DB00137	Osteitis Deformans	C0029401
Glycine	DB00145	Malignant Neoplasm of Lung	C0242379
Thiamine	DB00152	Exanthema	C0015230
Thiamine	DB00152	Legionella Pneumophila Pneumonia	C0857846
Thiamine	DB00152	Pneumococcal Infections	C0032269
Cholecalciferol	DB00169	Atrophic Vaginitis	C0221392
Cholecalciferol	DB00169	Adrenal Cortical Hypofunction	C0405580
Cholecalciferol	DB00169	Urinary Retention	C0080274
Pravastatin	DB00175	Conjunctivitis, Vernal	C0009773
Esmolol	DB00187	Decompensated Cardiac Failure	C0581377
Bortezomib	DB00188	Nasal Congestion (Finding)	C0027424
Carbidopa	DB00190	Haemophilus Parainfluenzae Pneumonia	C0877633
Phentermine	DB00191	Enterobacter Pneumonia	C1096258
Phentermine	DB00191	Tinea Capitis	C0040250
Phentermine	DB00191	Q Fever Endocarditis	C0340354
Tramadol	DB00193	Acute Gonococcal Cervicitis	C0153195
Tramadol	DB00193	Cardiac Arrest	C0018790
Vidarabine	DB00194	Tendinitis	C0039503
Fluconazole	DB00196	Onychomycosis of Toenails	C1274470
Fluconazole	DB00196	Cluster Headache	C0009088
Fluconazole	DB00196	Tinea Versicolor	C0040262
Fluconazole	DB00196	Diaper Rash	C0011974
Caffeine	DB00201	Anthrax Disease	C0003175

Continued on next page

Table 2 – continued from previous page

Drug name	DrugBank ID	Disease name	Disease CUI
Caffeine	DB00201	Muscle Spasticity of Spinal Origin	C1562430
Azithromycin	DB00207	Malignant Lymphoma, Lymphocytic, Intermediate Differentiation, Diffuse	C0334634
Azithromycin	DB00207	Escherichia Coli Septicemia	C0276088
Azithromycin	DB00207	Diabetic Foot Infection	C0744130
Moxifloxacin	DB00218	Chronic Pain	C0150055
Moxifloxacin	DB00218	Epilepsies, Partial	C0014547
Moxifloxacin	DB00218	Pruritus Ani	C0033775
Moxifloxacin	DB00218	Poisoning By Pyrimethamine	C0274478
Methyclothiazide	DB00232	Angina Pectoris	C0002962
Aminosalicylic Acid	DB00233	Erythema Nodosum Leprosum	C0343467
Aminosalicylic Acid	DB00233	Nausea and Vomiting	C0027498
Mesalazine	DB00244	Furunculosis	C0016867
Clotrimazole	DB00257	Blast Phase	C0005699
Sulfanilamide	DB00259	Pneumococcal Infections	C0032269
Cycloserine	DB00260	Streptococcal Tonsillitis	C0275804
Sulfisoxazole	DB00263	Keratoconjunctivitis	C0022573
Sulfisoxazole	DB00263	Streptococcus Pyogenes Infection	C0554628
Crotamiton	DB00265	Chlamydial Cervicitis	C0341834
Crotamiton	DB00265	Degenerative Polyarthritis	C0029408
Crotamiton	DB00265	Bursitis	C0006444
Crotamiton	DB00265	Herpes Zoster Keratitis	C1275687
Crotamiton	DB00265	Congenital Hemolytic Uremic Syndrome	C2919522
Topiramate	DB00273	Sneezing	C0037383
Topiramate	DB00273	Granuloma Annulare	C0085074
Topiramate	DB00273	Staphylococcus Aureus Infection	C1318973
Topiramate	DB00273	Tinea Corporis (Disorder)	C0040252
Topiramate	DB00273	Rosacea	C0035854
Cefmetazole	DB00274	Muscle Spasticity of Spinal Origin	C1562430
Clemastine	DB00283	Psychotic Disorders	C0033975
Penciclovir	DB00299	Urinary Tract Infection Enterococcus	C0749958
Penciclovir	DB00299	Fusariosis	C0276758
Tenofovir	DB00300	Chronic Small Plaque Psoriasis	C0406317
Tranexamic Acid	DB00302	Synovitis	C0039103
Ertapenem	DB00303	Anemia of Prematurity	C0158996
Ertapenem	DB00303	Septicemia Due to Bacteroides	C0276064
Chlorthalidone	DB00310	Haemophilus Parainfluenzae Pneumonia	C0877633
Chlorthalidone	DB00310	Pneumonia, Bacterial	C0004626
Chlorthalidone	DB00310	Rickettsia Infections	C0035585
Capreomycin	DB00314	Haemophilus Influenzae Type B Infection	C2028293
Codeine	DB00318	Epicondylitis	C0014488
Piperacillin	DB00319	Carbamoyl-Phosphate Synthase I Deficiency Disease	C0751753
Floxuridine	DB00322	Cutaneous Candidiasis	C0006846

Continued on next page

Table 2 – continued from previous page

Drug name	DrugBank ID	Disease name	Disease CUI
Nitroprusside	DB00325	Acute Lymphocytic Leukemia	C0023449
Indomethacin	DB00328	Gingivostomatitis	C0149704
Indomethacin	DB00328	Contact Dermatitis	C0011616
Ipratropium bromide	DB00332	Acute Bacterial Sinusitis	C0275556
Nitrofural	DB00336	Chemotherapy-Induced Nausea and Vomiting	C0401160
Cetirizine	DB00341	Impetigo	C0021099
Minoxidil	DB00350	Hypoparathyroidism	C0020626
Aztreonam	DB00355	Nosocomial Pneumonia	C0949083
Aztreonam	DB00355	Exacerbation of Asthma	C0349790
Aztreonam	DB00355	Severe Pain	C0278140
Aztreonam	DB00355	Chemotherapy-Induced Nausea and Vomiting	C0401160
Aztreonam	DB00355	Acute Bacterial Bronchitis	C0339933
Aztreonam	DB00355	Sinusitis	C0037199
Doxylamine	DB00366	Hypotension	C0020649
Doxylamine	DB00366	Complex Dyslipidemia	C3875286
Levonorgestrel	DB00367	Onychomycosis	C0040261
Norepinephrine	DB00368	Escherichia Coli Urinary Tract Infection	C0577708
Colestipol	DB00375	Rhinitis	C0035455
Colestipol	DB00375	Fever	C0015967
Carbimazole	DB00389	Scurfiness of Scalp	C0423775
Digoxin	DB00390	Enterobacter Pneumonia	C1096258
Digoxin	DB00390	Simple Partial Seizures	C0234974
Beclomethasone dipropionate	DB00394	Dermatologic Disorders	C0037274
Beclomethasone dipropionate	DB00394	Tinea Versicolor	C0040262
Progesterone	DB00396	Shigella Infections	C0013371
Griseofulvin	DB00400	Chlamydial Pneumonia	C0339959
Griseofulvin	DB00400	Genitourinary Tract Infection	C1279247
Dexbrompheniramine	DB00405	Pharyngitis	C0031350
Ampicillin	DB00415	Hypotension	C0020649
Zolpidem	DB00425	Lupus Erythematosus, Discoid	C0024138
Zolpidem	DB00425	Infection Due to Pseudomonas Aeruginosa	C0276075
Carboprost Tromethamine	DB00429	Plague	C0032064
Cyproheptadine	DB00434	Nocardia Infections	C0028242
Allopurinol	DB00437	Psoriasis	C0033860
Ceftazidime	DB00438	Vitamin D-Dependent Rickets	C0221468
Ceftazidime	DB00438	Kaposi Sarcoma	C0036220
Ceftazidime	DB00438	Fever	C0015967
Trimethoprim	DB00440	Familial Hypercholesterolemia - Homozygous	C0342881
Betamethasone	DB00443	Meningitis	C0025289
Betamethasone	DB00443	Mycoplasma Pneumonia	C0032302
Betamethasone	DB00443	Metrorrhagia	C0025874

Continued on next page

Table 2 – continued from previous page

Drug name	DrugBank ID	Disease name	Disease CUI
Betamethasone	DB00443	Postherpetic Neuralgia	C0032768
Betamethasone	DB00443	Arthropathy Associated With Infection	C0157749
Betamethasone	DB00443	Poisoning By Sulfadiazine	C0274462
Pethidine	DB00454	Diarrhea	C0011991
Loratadine	DB00455	Salmonella Sepsis	C0152486
Cefalotin	DB00456	Renal Cell Carcinoma	C0007134
Cefalotin	DB00456	Glaucoma, Open-Angle	C0017612
Cefalotin	DB00456	Pediculus Capitis Infestation	C0030757
Cefalotin	DB00456	Gonococcal Infection Disseminated	C0744451
Cefalotin	DB00456	Anemia of Prematurity	C0158996
Cefalotin	DB00456	Urgency of Micturition	C0085606
Chlordiazepoxide	DB00475	Lennox-Gastaut Syndrome	C0238111
Duloxetine	DB00476	Multiple Sclerosis	C0026769
Chlorpromazine	DB00477	Malignant Essential Hypertension	C0024588
Amikacin	DB00479	Haemophilus Influenzae Type B Infection	C2028293
Amikacin	DB00479	Sore Throat	C0242429
Celecoxib	DB00482	Erysipelas	C0014733
Dicloxacillin	DB00485	Malignant Lymphoma, Lymphocytic, Intermediate Differentiation, Diffuse	C0334634
Cefotaxime	DB00493	Acne Vulgaris	C0001144
Cefotaxime	DB00493	Multiple Endocrine Neoplasia	C0027662
Cefotaxime	DB00493	Peripheral Vascular Diseases	C0085096
Nitazoxanide	DB00507	Severe Pain	C0278140
Nitazoxanide	DB00507	Transplanted Organ Rejection	C0345468
Vancomycin	DB00512	Syphilis, Latent	C0039133
Vancomycin	DB00512	Citrullinemia	C0175683
Vancomycin	DB00512	Serum Sickness	C0036830
Dextromethorphan	DB00514	Status Asthmaticus	C0038218
Ciprofloxacin	DB00537	Meningococcus Carrier	C0421162
Ciprofloxacin	DB00537	Mucositis Following Radiation Therapy	C0392190
Ciprofloxacin	DB00537	Sepsis Due to Staphylococcus Aureus	C2887088
Vincristine	DB00541	Cutaneous Candidiasis	C0006846
Propylthiouracil	DB00550	Chlamydia Trachomatis Infection of Genital Structure	C1997322
Methoxsalen	DB00553	Acute Moraxella Catarrhalis Bronchitis	C0339932
Methoxsalen	DB00553	Chronic Bacterial Prostatitis	C1720797
Carbamazepine	DB00564	Greasy Skin	C0234925
Cephalexin	DB00567	Renal Osteodystrophy	C0035086
Cephalexin	DB00567	Greasy Skin	C0234925
Cephalexin	DB00567	Follicular Thyroid Carcinoma	C0206682
Clonidine	DB00575	Chlamydia Trachomatis Infection of Genital Structure	C1997322
Sulfamethizole	DB00576	Lymphoma, Follicular	C0024301
Sulfamethizole	DB00576	Injury Wounds	C0043250
Sulfamethizole	DB00576	Onychomycosis	C0040261

Continued on next page

Table 2 – continued from previous page

Drug name	DrugBank ID	Disease name	Disease CUI
Lactulose	DB00581	Infection Due to Pseudomonas Aeruginosa	C0276075
Lactulose	DB00581	Humoral Hypercalcemia of Malignancy (Disorder)	C0149911
Lactulose	DB00581	Pruritus	C0033774
Voriconazole	DB00582	Allergic Rhinitis (Disorder)	C2607914
Fluocinolone Acetonide	DB00591	Moraxella Catarrhalis Pneumonia	C0857831
Oxytetracycline	DB00595	Drug-Induced Mucositis	C1274988
Oxytetracycline	DB00595	Breastfeeding (Mother)	C1623040
Oxytetracycline	DB00595	Septicemia Due to Enterococcus	C0588233
Ulobetasol	DB00596	Testicular Germ Cell Tumor	C1336708
Ulobetasol	DB00596	Anemia, Diamond-Blackfan	C1260899
Ulobetasol	DB00596	Parkinson Disease	C0030567
Ulobetasol	DB00596	Malignant Lymphoma, Lymphocytic, Intermediate Differentiation, Diffuse	C0334634
Ulobetasol	DB00596	Chronic Tubotympanic Suppurative Otitis Media	C0155440
Ulobetasol	DB00596	Cutaneous Anthrax	C0003177
Nafcillin	DB00607	Lower Respiratory Tract Infection	C0149725
Butorphanol	DB00611	Yaws	C0043388
Butorphanol	DB00611	Otitis Externa	C0029878
Butorphanol	DB00611	Common Cold	C0009443
Demeclocycline	DB00618	Acquired Partial Lipodystrophy	C0220989
Imatinib	DB00619	Enterobacter Pneumonia	C1096258
Triamcinolone	DB00620	Impetigo	C0021099
Triamcinolone	DB00620	Osteoporosis	C0029456
Triamcinolone	DB00620	Coughing	C0010200
Triamcinolone	DB00620	Gonococcal Joint Infection	C0153216
Triamcinolone	DB00620	Obesity	C0028754
Triamcinolone	DB00620	Multiple Myeloma	C0026764
Niacin	DB00627	Bacterial Conjunctivitis	C0009768
Sulfacetamide	DB00634	Muscle Spasticity of Spinal Origin	C1562430
Prednisone	DB00635	Nausea and Vomiting	C0027498
Prednisone	DB00635	Vomiting	C0042963
Prednisone	DB00635	Mineral Deficiency	C0687148
Prednisone	DB00635	Diabetic Foot Infection	C0744130
Prednisone	DB00635	Infection Due to Pseudomonas Aeruginosa	C0276075
Astemizole	DB00637	Parkinsonian Disorders	C0242422
Simvastatin	DB00641	Seborrheic Dermatitis	C0036508
Simvastatin	DB00641	Acute Promyelocytic Leukemia	C0023487
Simvastatin	DB00641	Bronchitis, Chronic	C0008677
Leucovorin	DB00650	Endemic Flea-Borne Typhus	C0041472
Pentazocine	DB00652	Acute Promyelocytic Leukemia	C0023487
Verapamil	DB00661	Female Hypogonadism Syndrome	C0271578
Verapamil	DB00661	Attention Deficit Hyperactivity Disorder	C1263846
Trimethobenzamide	DB00662	Blast Phase	C0005699

Continued on next page

Table 2 – continued from previous page

Drug name	DrugBank ID	Disease name	Disease CUI
Trimethobenzamide	DB00662	Yaws	C0043388
Cefixime	DB00671	Lower Respiratory Tract Infection	C0149725
Amphotericin B	DB00681	Cycloplegia	C0235238
Amphotericin B	DB00681	Irritable Bowel Syndrome	C0022104
Midazolam	DB00683	Rosacea	C0035854
Trovafloxacin	DB00685	Itching of Eye	C0022281
Trovafloxacin	DB00685	Aspiration Pneumonitis	C1761609
Trovafloxacin	DB00685	Rhinitis	C0035455
Fludrocortisone	DB00687	Skin Diseases, Bacterial	C0162627
Fludrocortisone	DB00687	Thyroiditis	C0040147
Fludrocortisone	DB00687	Interstitial Cystitis	C0282488
Fludrocortisone	DB00687	Rosacea	C0035854
Daunorubicin	DB00694	Haemophilus Parainfluenzae Pneumonia	C0877633
Daunorubicin	DB00694	Degenerative Polyarthritis	C0029408
Daunorubicin	DB00694	Lymphoma, T-Cell, Cutaneous	C0079773
Daunorubicin	DB00694	Chronic Idiopathic Urticaria	C0578870
Tizanidine	DB00697	Acute Promyelocytic Leukemia	C0023487
Nitrofurantoin	DB00698	Allergic Rhinitis (Disorder)	C2607914
Nitrofurantoin	DB00698	Sore Throat	C0242429
Naltrexone	DB00704	Rhinitis, Vasomotor	C0035460
Porfimer	DB00707	Pharyngitis Due to Haemophilus Influenzae	C2062475
Porfimer	DB00707	Thyroiditis	C0040147
Lamivudine	DB00709	Postoperative Infection	C0392618
Ibandronate	DB00710	Headache Disorders	C0393735
Oxacillin	DB00713	Hyperkeratosis	C0870082
Oxacillin	DB00713	Streptococcal Sepsis	C0152964
Azatadine	DB00719	Hodgkin Disease	C0019829
Risperidone	DB00734	Scalp Psoriasis	C0406326
Esomeprazole	DB00736	Papillary Thyroid Carcinoma	C0238463
Esomeprazole	DB00736	Lymphoma, T-Cell, Cutaneous	C0079773
Esomeprazole	DB00736	Candidiasis	C0006840
Esomeprazole	DB00736	Nausea	C0027497
Hydrocortisone	DB00741	Gonorrhea of Pharynx	C0149966
Hydrocortisone	DB00741	Malignant Tumor of Peritoneum	C0153467
Hydrocortisone	DB00741	Empyema, Pleural	C0014013
Hydrocortisone	DB00741	Granuloma Inguinale	C0018190
Scopolamine	DB00747	Bacterial Keratitis	C0854211
Epinastine	DB00751	Impetigo	C0021099
Epinastine	DB00751	Abdominal Abscess	C0243001
Ethotoin	DB00754	Uveitis	C0042164
Ethotoin	DB00754	Pelvic Inflammatory Disease	C0242172
Tretinoin	DB00755	Tetanus	C0039614
Tretinoin	DB00755	Chlamydial Urethritis	C1278807

Continued on next page

Table 2 – continued from previous page

Drug name	DrugBank ID	Disease name	Disease CUI
Meropenem	DB00760	Urinary Tract Infection	C0042029
Potassium Chloride	DB00761	Abdominal Abscess	C0243001
Mometasone	DB00764	Muscle Spasticity	C0026838
Alprostadil	DB00770	Lower Respiratory Tract Infection	C0149725
Tirofiban	DB00775	Neonatal Meningitis	C0456107
Polymyxin B Sulfate	DB00781	Septicemia Due to Bacteroides	C0276064
Mefenamic acid	DB00784	Angioedema	C0002994
Mefenamic acid	DB00784	Gonorrhea	C0018081
Naproxen	DB00788	Lymphoma, Follicular	C0024301
Tripelennamine	DB00792	Pyelonephritis E Coli	C0748196
Gentamicin	DB00798	Labor Pain	C0474368
Gentamicin	DB00798	Polycystic Kidney, Autosomal Dominant	C0085413
Gentamicin	DB00798	Osteitis Deformans	C0029401
Gentamicin	DB00798	Tinea Barbae	C2349994
Fenoldopam	DB00800	Hypercholesterolemia	C0020443
Fenoldopam	DB00800	Uric Acid Renal Calculus	C0558595
Colistin	DB00803	Sepsis Due to Pseudomonas	C2887096
Biperiden	DB00810	Endometriosis	C0014175
Menthol	DB00825	Meningococcal Meningitis	C0025294
Fosfomycin	DB00828	Rhinoscleroma	C0035468
Diazepam	DB00829	Vitamin B 12 Deficiency	C0042847
Diazepam	DB00829	Deficiency of Testosterone Biosynthesis	C0342527
Cefaclor	DB00833	Scarlet Fever	C0036285
Nalbuphine	DB00844	Follicular Thyroid Carcinoma	C0206682
Nalbuphine	DB00844	Uric Acid Renal Calculus	C0558595
Nalbuphine	DB00844	Tenosynovitis	C0039520
Clofazimine	DB00845	Enterobacter Pneumonia	C1096258
Temozolomide	DB00853	Lymphoma, Non-Hodgkin	C0024305
Terbinafine	DB00857	Q Fever Endocarditis	C0340354
Terbinafine	DB00857	Acute Gonococcal Cervicitis	C0153195
Penicillamine	DB00859	Diarrhoea Predominant Irritable Bowel Syndrome	C1262211
Penicillamine	DB00859	Coughing	C0010200
Prednisolone	DB00860	Symptomatic Dermographism	C0343065
Prednisolone	DB00860	Constipation	C0009806
Prednisolone	DB00860	Pruritus	C0033774
Prednisolone	DB00860	Muscle Spasticity of Spinal Origin	C1562430
Terbutaline	DB00871	Malignant Otitis Externa Due to Pseudomonas Aeruginosa	C0395818
Terbutaline	DB00871	Infection of Bone	C2242472
Loteprednol	DB00873	Tinea Cruris	C1384589
Loteprednol	DB00873	Streptococcal Pneumonia	C0155862
Loteprednol	DB00873	Sepsis Due to Staphylococcus Aureus	C2887088
Loteprednol	DB00873	Dry Eye Syndromes	C0013238

Continued on next page

Table 2 – continued from previous page

Drug name	DrugBank ID	Disease name	Disease CUI
Loteprednol	DB00873	Drug-Induced Mucositis	C1274988
Loteprednol	DB00873	Yaws	C0043388
Loteprednol	DB00873	Relapsing Fever	C0035021
Guaifenesin	DB00874	Chronic Bacterial Prostatitis	C1720797
Guaifenesin	DB00874	Gout	C0018099
Sirolimus	DB00877	Psoriasis	C0033860
Isosorbide Dinitrate	DB00883	Tinea Corporis (Disorder)	C0040252
Pemirolast	DB00885	Infection Due to Pseudomonas Aeruginosa	C0276075
Dienestrol	DB00890	Anxiety	C0003467
Dienestrol	DB00890	Angioedema	C0002994
Methdilazine	DB00902	Urinary Tract Infection Citrobacter	C0749955
Amantadine	DB00915	Staphylococcal Pneumonia	C0032308
Metronidazole	DB00916	Labor Pain	C0474368
Dinoprostone	DB00917	Chlamydia Trachomatis Infection of Genital Structure	C1997322
Buprenorphine	DB00921	Rhinorrhea	C1260880
Buprenorphine	DB00921	Adrenal Cortical Hypofunction	C0405580
Maprotiline	DB00934	Symptomatic Dermographism	C0343065
Salicylic acid	DB00936	Klebsiella Sepsis	C0745528
Phenprocoumon	DB00946	Peptic Ulcer	C0030920
Mezlocillin	DB00948	Chlamydia Trachomatis Infection of Genital Structure	C1997322
Mezlocillin	DB00948	Deficiency of Testosterone Biosynthesis	C0342527
Dirithromycin	DB00954	Metastasis From Malignant Tumor of Colon	C1282500
Hydrocodone	DB00956	Urinary Tract Infection	C0042029
Methylprednisolone	DB00959	Raynaud Disease	C0034734
Methylprednisolone	DB00959	Acute Exacerbation of Chronic Bronchitis	C0856695
Methylprednisolone	DB00959	Asymptomatic Left Ventricular Systolic Dysfunction	C3698411
Zaleplon	DB00962	Labor Pain	C0474368
Desloratadine	DB00967	Genitourinary Tract Infection	C1279247
Alosetron	DB00969	Proteus Urinary Tract Infection	C0577709
Alosetron	DB00969	Glioblastoma Multiforme of Brain	C0349543
Selenium Sulfide	DB00971	Granuloma Inguinale	C0018190
Edetic Acid	DB00974	Blast Phase	C0005699
Dipyridamole	DB00975	Trachoma	C0040592
Formoterol	DB00983	Common Cold	C0009443
Dimenhydrinate	DB00985	Myocardial Infarction	C0027051
Rivastigmine	DB00989	Bronchitis	C0006277
Neomycin	DB00994	Pneumonia Due to Escherichia Coli	C0276089
Gabapentin	DB00996	Epicondylitis	C0014488
Doxorubicin	DB00997	Malaria, Vivax	C0024537
Doxorubicin	DB00997	Chronic Idiopathic Constipation	C0267509
Doxorubicin	DB00997	Osteomalacia	C0029442
Hydrochlorothiazide	DB00999	Rhinitis, Vasomotor	C0035460
Ganciclovir	DB01004	Anemia, Pernicious	C0002892

Continued on next page

Table 2 – continued from previous page

Drug name	DrugBank ID	Disease name	Disease CUI
Ganciclovir	DB01004	Muscle Spasticity of Spinal Origin	C1562430
Clobetasol propionate	DB01013	Herpes Zoster Keratitis	C1275687
Sulfamethoxazole	DB01015	Allergic Otitis Externa	C1320547
Sulfamethoxazole	DB01015	Onychomycosis	C0040261
Sulfamethoxazole	DB01015	Iridocyclitis	C0022073
Sulfamethoxazole	DB01015	Syphilis, Latent	C0039133
Minocycline	DB01017	Lichen Simplex Chronicus	C0149922
Minocycline	DB01017	Acute Bacterial Sinusitis	C0275556
Minocycline	DB01017	Zollinger-Ellison Syndrome	C0043515
Minocycline	DB01017	Septicemia Due to Enterococcus	C0588233
Minocycline	DB01017	Erythema Multiforme	C0014742
Guanfacine	DB01018	Epilepsy Characterized By Intractable Complex Partial Seizures	C2711653
Topotecan	DB01030	Serratia Sepsis	C0152973
Mercaptopurine	DB01033	Sarcoidosis	C0036202
Selegiline	DB01037	Nasal Congestion (Finding)	C0027424
Selegiline	DB01037	Osteomalacia	C0029442
Thalidomide	DB01041	Pharyngitis	C0031350
Fluocinonide	DB01047	Myelofibrosis	C0026987
Fluocinonide	DB01047	Malignant Ascites	C0220656
Ibuprofen	DB01050	Common Cold	C0009443
Ibuprofen	DB01050	Renal Disease With Edema Nos	C0866125
Benzylpenicillin	DB01053	Rosacea	C0035854
Benzylpenicillin	DB01053	Malignant Neoplasm of Ovary	C1140680
Praziquantel	DB01058	Bacterial Conjunctivitis	C0009768
Norfloxacin	DB01059	Central Retinal Vein Occlusion With Macular Edema	C0339498
Norfloxacin	DB01059	Pemphigus	C0030807
Norfloxacin	DB01059	Extrapyramidal Disorders	C0015371
Amoxicillin	DB01060	Anthrax Disease	C0003175
Amoxicillin	DB01060	Irritable Bowel Syndrome	C0022104
Amoxicillin	DB01060	Aspergillosis	C0004030
Amoxicillin	DB01060	Chronic Obstructive Airway Disease	C0024117
Azlocillin	DB01061	Lung Abscess	C0024110
Azlocillin	DB01061	Metastatic Prostate Carcinoma	C0936223
Cefditoren	DB01066	Hepatic Coma	C0019147
Dihydrotachysterol	DB01070	Goiter	C0018021
Etidronic acid	DB01077	Urinary Tract Infection Enterococcus	C0749958
Vigabatrin	DB01080	Beta Thalassemia	C0005283
Vigabatrin	DB01080	Vascular Diseases	C0042373
Streptomycin	DB01082	Shigella Infections	C0013371
Streptomycin	DB01082	Genitourinary Tract Infection	C1279247
Pilocarpine	DB01085	Secondary Malignant Neoplasm of Stomach	C0686068
Pilocarpine	DB01085	Septicemia Candida	C0349009

Continued on next page

Table 2 – continued from previous page

Drug name	DrugBank ID	Disease name	Disease CUI
Pilocarpine	DB01085	Granuloma Annulare	C0085074
Capecitabine	DB01101	Muscle Spasticity	C0026838
Sertraline	DB01104	Chronic Idiopathic Constipation	C0267509
Miconazole	DB01110	Chancroids	C0007947
Colistimethate	DB01111	Nasal Congestion (Finding)	C0027424
Cefuroxime	DB01112	Arthritis, Psoriatic	C0003872
Cefuroxime	DB01112	Thromboembolism	C0040038
Cefuroxime	DB01112	Arthropod Bite Wound	C1444173
Cefuroxime	DB01112	Pneumonia Due to Pseudomonas	C0155860
Cefuroxime	DB01112	Rectal Pain	C0034886
Diazoxide	DB01119	Degenerative Polyarthritis	C0029408
Phenacemide	DB01121	Acute Postoperative Pain	C2215257
Phenacemide	DB01121	Ocular Hypertension	C0028840
Rabeprazole	DB01129	Pneumonia Due to Escherichia Coli	C0276089
Rabeprazole	DB01129	Epilepsy Characterized By Intractable Complex Partial Seizures	C2711653
Levofloxacin	DB01137	Scurfiness of Scalp	C0423775
Levofloxacin	DB01137	Vomiting	C0042963
Levofloxacin	DB01137	Mixed Anxiety and Depressive Disorder	C0338908
Levofloxacin	DB01137	Lymphogranuloma Venereum	C0024286
Levofloxacin	DB01137	Echinococcus Granulosus Infection of Liver	C0153289
Levofloxacin	DB01137	Schistosomiasis	C0036323
Levofloxacin	DB01137	Malignant Neoplasm of Stomach Stage Iv	C0278498
Cefapirin	DB01139	Initial Insomnia	C0393760
Cefapirin	DB01139	Opisthorchiasis	C0029106
Cefapirin	DB01139	Malignant Neoplasm of Liver	C0345904
Cefapirin	DB01139	Serratia Sepsis	C0152973
Cefadroxil	DB01140	Injury Wounds	C0043250
Diphenylpyraline	DB01146	Breastfeeding (Mother)	C1623040
Flavoxate	DB01148	Multiple Myeloma	C0026764
Cefprozil	DB01150	Laryngeal Edema	C0023052
Cefprozil	DB01150	Staphylococcal Skin Infections	C0038166
Gemifloxacin	DB01155	Chronic Tubotympanic Suppurative Otitis Media	C0155440
Gemifloxacin	DB01155	Nosocomial Pneumonia	C0949083
Gemifloxacin	DB01155	Staphylococcus Aureus Infection	C1318973
Bretylium	DB01158	Angioedema	C0002994
Ofloxacin	DB01165	Urge Incontinence	C0150045
Ofloxacin	DB01165	Itching of Ear	C0849907
Ofloxacin	DB01165	Low Cardiac Output Syndrome	C0600177
Ofloxacin	DB01165	Scalp Dermatoses	C0036271
Ofloxacin	DB01165	Impetigo	C0021099
Itraconazole	DB01167	Primary Malignant Neoplasm of Gastrointestinal Tract	C1306632
Itraconazole	DB01167	Drug-Induced Mucositis	C1274988

Continued on next page

Table 2 – continued from previous page

Drug name	DrugBank ID	Disease name	Disease CUI
Itraconazole	DB01167	Low Cardiac Output Syndrome	C0600177
Arsenic trioxide	DB01169	Cutaneous Candidiasis	C0006846
Arsenic trioxide	DB01169	Rheumatic Heart Disease	C0035439
Arsenic trioxide	DB01169	Lymphoma, T-Cell, Cutaneous	C0079773
Arsenic trioxide	DB01169	Peritonitis	C0031154
Arsenic trioxide	DB01169	Arthritis, Gouty	C0003868
Orphenadrine	DB01173	Chemotherapy-Induced Nausea and Vomiting	C0401160
Escitalopram	DB01175	Mydriasis	C0026961
Escitalopram	DB01175	Chronic Pain	C0150055
Sparfloxacin	DB01208	Intractable Hiccups	C0744896
Sparfloxacin	DB01208	Pneumonia Due to Klebsiella Pneumoniae	C0519030
Sparfloxacin	DB01208	Salmonella Sepsis	C0152486
Sparfloxacin	DB01208	Urinary Retention	C0080274
Sparfloxacin	DB01208	Osteoporosis, Postmenopausal	C0029458
Sparfloxacin	DB01208	Dacryocystitis	C0010930
Sparfloxacin	DB01208	Uric Acid Renal Calculus	C0558595
Clarithromycin	DB01211	Acromegaly	C0001206
Clarithromycin	DB01211	Initial Insomnia	C0393760
Ceftriaxone	DB01212	Vomiting	C0042963
Ceftriaxone	DB01212	Onychomycosis of Fingernails	C1274469
Fomepizole	DB01213	Bacterial Infection Due to Klebsiella Pneumoniae	C0343402
Finasteride	DB01216	Allergic Conjunctivitis	C0009766
Finasteride	DB01216	Female Genital Tract Infection	C1263758
Dantrolene	DB01219	Corneal Perforation	C0339293
Dantrolene	DB01219	Dermatitis Herpetiformis	C0011608
Rifaximin	DB01220	Motion Sickness	C0026603
Rifaximin	DB01220	Pain	C0030193
Budesonide	DB01222	Enterobacteriaceae Infections	C0014347
Metoclopramide	DB01233	Tetanus	C0039614
Metoclopramide	DB01233	Escherichia Coli Urinary Tract Infection	C0577708
Metoclopramide	DB01233	Acute Gonococcal Epididymo-Orchitis	C0153193
Dexamethasone	DB01234	Endometriosis	C0014175
Dexamethasone	DB01234	Hereditary Orotic Aciduria	C0220987
Dexamethasone	DB01234	Hepatitis C, Chronic	C0524910
Dexamethasone	DB01234	Mineral Deficiency	C0687148
Dexamethasone	DB01234	Urinary Tract Infection	C0042029
Dexamethasone	DB01234	Hepatolenticular Degeneration	C0019202
Dexamethasone	DB01234	Q Fever Endocarditis	C0340354
Epoprostenol	DB01240	Redness of Eye	C0235267
Epoprostenol	DB01240	Edema	C0013604
Alimemazine	DB01246	Tetanus	C0039614
Docetaxel	DB01248	Female Hypogonadism Syndrome	C0271578
Eculizumab	DB01257	Lower Respiratory Tract Infection	C0149725

Continued on next page

Table 2 – continued from previous page

Drug name	DrugBank ID	Disease name	Disease CUI
Eculizumab	DB01257	Motion Sickness	C0026603
Eculizumab	DB01257	Congestive Heart Failure	C0018802
Eculizumab	DB01257	Osteitis Deformans	C0029401
Desonide	DB01260	Metastasis From Malignant Tumor of Colon	C1282500
Desonide	DB01260	Autoimmune Hemolytic Anemia	C0002880
Desonide	DB01260	Chronic Lymphocytic Leukemia	C0023434
Desonide	DB01260	Pyelonephritis E Coli	C0748196
Sinecatechins	DB01266	Typhus	C0041471
Sinecatechins	DB01266	Bartonella Infections	C0004771
Sinecatechins	DB01266	Labor Pain	C0474368
Sinecatechins	DB01266	Ankylosing Spondylitis	C0038013
Sinecatechins	DB01266	Escherichia Coli Septicemia	C0276088
Sinecatechins	DB01266	Gonococcal Infection Disseminated	C0744451
Hydralazine	DB01275	Acute Gonococcal Cervicitis	C0153195
Carbetocin	DB01282	Infection of Skin And/Or Subcutaneous Tissue	C0178299
Bismuth Subsalicylate	DB01294	Trachoma	C0040592
Cefamandole	DB01326	Atrophic Vaginitis	C0221392
Cefamandole	DB01326	Typhus, Epidemic Louse-Borne	C0041473
Cefamandole	DB01326	Irritable Bowel Syndrome	C0022104
Cefamandole	DB01326	Dacryocystitis	C0010930
Cefazolin	DB01327	Septicemia Due to Enterococcus	C0588233
Cefazolin	DB01327	Psoriasis	C0033860
Cefazolin	DB01327	Chlamydial Pneumonia	C0339959
Cefotetan	DB01330	Ethylene Glycol Poisoning (Disorder)	C0413194
Cefotetan	DB01330	Impetigo	C0021099
Cefotetan	DB01330	Initial Insomnia	C0393760
Cefotetan	DB01330	Recurrent Herpes Simplex Labialis	C1274321
Cefoxitin	DB01331	Erectile Dysfunction	C0242350
Cefoxitin	DB01331	Body Louse Infestation	C0030758
Cefoxitin	DB01331	Accidental Poisoning By Methyl Alcohol	C0261439
Cefoxitin	DB01331	Nocardia Infections	C0028242
Ceftizoxime	DB01332	Postherpetic Neuralgia	C0032768
Cefradine	DB01333	Listeriosis	C0023860
Cefradine	DB01333	Meningococcal Meningitis	C0025294
Lithium	DB01356	Chemotherapy-Induced Nausea and Vomiting	C0401160
Magnesium oxide	DB01377	Drug-Induced Mucositis	C1274988
Cortisone acetate	DB01380	Allergic Otitis Externa	C1320547
Cortisone acetate	DB01380	Acute Amebiasis	C0152499
Cortisone acetate	DB01380	Boutonneuse Fever	C0006060
Cortisone acetate	DB01380	Greasy Skin	C0234925
Cortisone acetate	DB01380	Obesity	C0028754
Magnesium salicylate	DB01397	Pneumonia Due to Staphylococcus Aureus	C2349530
Cefepime	DB01413	Adenocarcinoma of Pancreas	C0281361

Continued on next page

Table 2 – continued from previous page

Drug name	DrugBank ID	Disease name	Disease CUI
Ceftibuten	DB01415	Typhus, Epidemic Louse-Borne	C0041473
Ceftibuten	DB01415	Epilepsy	C0014544
Ceftibuten	DB01415	Epicondylitis	C0014488
Ceftibuten	DB01415	Arthropathy Associated With Infection	C0157749
Paromomycin	DB01421	Epilepsies, Myoclonic	C0014550
Paromomycin	DB01421	Secondary Physiologic Amenorrhea	C0232940
Paromomycin	DB01421	Actinic Keratosis	C0022602
Ethyl loflazepate	DB01545	Hypertensive Disease	C0020538
Ethyl loflazepate	DB01545	Pneumonia Due to Staphylococcus Aureus	C2349530
Dihydrocodeine	DB01551	Accidental Poisoning By Methyl Alcohol	C0261439
Dihydrocodeine	DB01551	Enteric Campylobacteriosis	C0275982
Dihydrocodeine	DB01551	Breastfeeding (Mother)	C1623040
Dextroamphetamine	DB01576	Rhinorrhea	C1260880
Oxprenolol	DB01580	Chronic Idiopathic Urticaria	C0578870
Oxprenolol	DB01580	Diarrhea	C0011991
Everolimus	DB01590	Allergic Conjunctivitis	C0009766
Cilastatin	DB01597	Rhinorrhea	C1260880
Cilastatin	DB01597	Mixed Bipolar I Disorder	C0236780
Bacampicillin	DB01602	Enterobiasis	C0086227
Bacampicillin	DB01602	Parkinsonian Disorders	C0242422
Bacampicillin	DB01602	Bacterial Vaginosis	C0085166
Bacampicillin	DB01602	Exanthema	C0015230
Tazobactam	DB01606	Blast Phase	C0005699
Ticarcillin	DB01607	Paronychia Inflammation	C0030578
Ticarcillin	DB01607	Paroxysmal Nocturnal Hemoglobinuria	C0024790
Ticarcillin	DB01607	Secondary Malignant Neoplasm of Pancreas	C0346976
Ticarcillin	DB01607	Chancroids	C0007947
Deferasirox	DB01609	Skin Irritation	C0152030
Deferasirox	DB01609	Lichen Simplex Chronicus	C0149922
Hydroxychloroquine	DB01611	Diabetic Macular Edema	C0730285
Phenindamine	DB01619	Sore Throat	C0242429
Pheniramine	DB01620	Non-Small Cell Lung Carcinoma	C0007131
Fusidic Acid	DB02703	Drug-Induced Mucositis	C1274988
Phenol	DB03255	Incomplete Passage of Stool	C0426639
Phenol	DB03255	Mucormycosis	C0026718
Phenol	DB03255	Chlamydial Pneumonia	C0339959
Phenol	DB03255	Hypophosphatemia	C0085682
Urea	DB03904	Rhinitis	C0035455
Trioxsalen	DB04571	Listeriosis	C0023860
Trioxsalen	DB04571	Trachoma	C0040592
Trioxsalen	DB04571	Greasy Skin	C0234925
Quinestrol	DB04575	Abdominal Abscess	C0243001
Clofedanol	DB04837	Musculoskeletal Diseases	C0026857

Continued on next page

Table 2 – continued from previous page

Drug name	DrugBank ID	Disease name	Disease CUI
Nilotinib	DB04868	Urinary Tract Infection	C0042029
Nesiritide	DB04899	Psittacosis	C0029291
Ustekinumab	DB05679	Pneumonia Due to Klebsiella Pneumoniae	C0519030
Acetylcysteine	DB06151	Bedwetting	C0270327
Canakinumab	DB06168	Onychomycosis Due to Trichophyton Mentagrophytes	C0276755
Romidepsin	DB06176	Arthropod Bite Wound	C1444173
Romidepsin	DB06176	Zollinger-Ellison Syndrome	C0043515
Asenapine	DB06216	Malignant Lymphoma, Lymphocytic, Intermediate Differentiation, Diffuse	C0334634
Dalbavancin	DB06219	Bacterial Conjunctivitis	C0009768
Ziconotide	DB06283	Lymphogranuloma Venereum	C0024286
Ziconotide	DB06283	Adrenogenital Disorder	C0701163
Temsirolimus	DB06287	Calcium Renal Calculus	C1959799
Temsirolimus	DB06287	Complex Dyslipidemia	C3875286
Temsirolimus	DB06287	Chemotherapy-Induced Nausea and Vomiting	C0401160
Temsirolimus	DB06287	Bacterial Urinary Infection	C0729524
Temsirolimus	DB06287	Simple Pulmonary Eosinophilia	C0242459
Telavancin	DB06402	Supraventricular Tachycardia	C0039240
Telavancin	DB06402	Boutonneuse Fever	C0006060
Telavancin	DB06402	Incomplete Passage of Stool	C0426639
Telavancin	DB06402	Inhalational Anthrax	C0155866
Ceftaroline fosamil	DB06590	Oral Candidiasis	C0006849
Ceftaroline fosamil	DB06590	Nausea	C0027497
Denosumab	DB06643	Rhinitis	C0035455
Denosumab	DB06643	Skin Irritation	C0152030
Mepyramine	DB06691	Blast Phase	C0005699
Xylometazoline	DB06694	Oropharyngeal Candidiasis	C0919659
Xylometazoline	DB06694	Female Genital Tract Infection	C1263758
Naphazoline	DB06711	Oropharyngeal Candidiasis	C0919659
Glycine betaine	DB06756	Scabies <Infestation>	C0036262
Glycine betaine	DB06756	Fever	C0015967
Ammonium lactate	DB06768	Mycoplasma Pneumonia	C0032302
Ammonium lactate	DB06768	Septicemia Due to Enterococcus	C0588233
Benzyl alcohol	DB06770	Hodgkin Disease	C0019829
Dimercaprol	DB06782	Chronic Obstructive Airway Disease	C0024117
Dimercaprol	DB06782	Anemia, Pernicious	C0002892
Dimercaprol	DB06782	Psittacosis	C0029291
Lanreotide	DB06791	Pruritus Ani	C0033775
Lodoxamide	DB06794	Blast Phase	C0005699
Lodoxamide	DB06794	Glaucoma, Open-Angle	C0017612
Niclosamide	DB06803	Rickettsialpox	C0035597
Sodium phenylbutyrate	DB06819	Pneumonia Due to Pseudomonas	C0155860
Antazoline	DB08799	Nocardia Infections	C0028242

Continued on next page

Table 2 – continued from previous page

Drug name	DrugBank ID	Disease name	Disease CUI
Roxatidine acetate	DB08806	Duodenal Ulcer	C0013295
Ticagrelor	DB08816	Cholera	C0008354
Aflibercept	DB08885	Scurfiness of Scalp	C0423775
Linaclotide	DB08890	Inclusion Conjunctivitis	C0009770
Linaclotide	DB08890	Sarcoidosis	C0036202
Linaclotide	DB08890	Decompensated Cardiac Failure	C0581377
Linaclotide	DB08890	Paroxysmal Nocturnal Hemoglobinuria	C0024790
Bedaquiline	DB08903	Coughing	C0010200
Certolizumab pegol	DB08904	Scalp Psoriasis	C0406326
Riociguat	DB08931	Bacterial Keratitis	C0854211
Riociguat	DB08931	Anemia, Diamond-Blackfan	C1260899
Riociguat	DB08931	Impetigo	C0021099
Riociguat	DB08931	Hay Fever	C0018621
Luliconazole	DB08933	Scalp Psoriasis	C0406326
Chlorcyclizine	DB08936	Proteus Septicemia	C0577690
Chlorcyclizine	DB08936	Bursitis	C0006444
Chlorcyclizine	DB08936	Rocky Mountain Spotted Fever	C0035793
Isoxsuprine	DB08941	Motion Sickness	C0026603
Vedolizumab	DB09033	Plague	C0032064
Efinaconazole	DB09040	Familial Hypercholesterolemia - Heterozygous	C0342882
Metreleptin	DB09046	Rosacea	C0035854
Ceftolozane	DB09050	Enterobacteriaceae Infections	C0014347
Avibactam	DB09060	Urinary Tract Infection	C0042029
Olaparib	DB09074	Syphilis, Congenital	C0039131
Lenvatinib	DB09078	Vomiting	C0042963
Lenvatinib	DB09078	Hypercalcemia	C0020437
Lenvatinib	DB09078	Endometritis	C0014179
Lenvatinib	DB09078	Infection Caused By Enterobacter	C0948205
Vilanterol	DB09082	Rickettsia Infections	C0035585
Vilanterol	DB09082	Iron Deficiency Anemia	C0162316
Vilanterol	DB09082	Inflammatory Dermatosis	C3875321
Vilanterol	DB09082	Rheumatic Heart Disease	C0035439
Vilanterol	DB09082	Rheumatoid Arthritis	C0003873
Vilanterol	DB09082	Bacterial Keratitis	C0854211
Benzoyl peroxide	DB09096	Gastrointestinal Anthrax	C0152945
Stiripentol	DB09118	Punctate Keratitis	C0259799
Stiripentol	DB09118	Acute Postoperative Pain	C2215257
Stiripentol	DB09118	Pruritus	C0033774
Stiripentol	DB09118	Laryngeal Edema	C0023052
Stiripentol	DB09118	Supraventricular Tachycardia	C0039240
Potassium Citrate	DB09125	Breastfeeding (Mother)	C1623040
Potassium Citrate	DB09125	Arteriosclerosis	C0003850
Uridine triacetate	DB09144	Allergic Conjunctivitis	C0009766

Continued on next page

Table 2 – continued from previous page

Drug name	DrugBank ID	Disease name	Disease CUI
Moxisylyte	DB09205	Non-Gonococcal Urethritis (Ngu)	C1112709
Dexibuprofen	DB09213	Syphilis	C0039128
Dexibuprofen	DB09213	Blastomycosis	C0005716
Dexibuprofen	DB09213	Thyroiditis	C0040147
Dexibuprofen	DB09213	Diabetic Foot Infection	C0744130
Dexibuprofen	DB09213	Tetanus	C0039614
Bemiparin	DB09258	Allergic Rhinitis (Disorder)	C2607914
Polyethylene glycol	DB09287	Gonorrhea of Rectum	C0275665
Alirocumab	DB09302	Sneezing	C0037383
Evolocumab	DB09303	Breastfeeding (Mother)	C1623040
Procaine benzylpenicillin	DB09320	Chronic Tubotympanic Suppurative Otitis Media	C0155440
Procaine benzylpenicillin	DB09320	Complex Dyslipidemia	C3875286
Zinc oxide	DB09321	Herpes Zoster Keratitis	C1275687
Zinc oxide	DB09321	Urinary Tract Infection Enterococcus	C0749958
Zinc oxide	DB09321	Ankylosing Spondylitis	C0038013
Zinc sulfate	DB09322	Brucellosis	C0006309
Zinc sulfate	DB09322	Lupus Erythematosus, Discoid	C0024138
Zinc sulfate	DB09322	Asthma	C0004096
Zinc sulfate	DB09322	Pneumonia, Bacterial	C0004626
Levobetaxolol	DB09351	Epicondylitis	C0014488
Sulfur	DB09353	Accidental Poisoning By Methyl Alcohol	C0261439
Sulfur	DB09353	Klebsiella Cystitis	C0520775
Dexpanthenol	DB09357	Acute Otitis Media	C0271429
Dexpanthenol	DB09357	Escherichia Coli Urinary Tract Infection	C0577708
Dexpanthenol	DB09357	Bartonella Infections	C0004771
Norgestrel	DB09389	Pneumonia, Bacterial	C0004626
Norgestrel	DB09389	Pelvic Inflammatory Disease	C0242172
Norgestrel	DB09389	Chickenpox	C0008049
Enalaprilat	DB09477	Mucositis Following Radiation Therapy	C0392190
Cysteine hydrochloride	DB09485	Epicondylitis	C0014488
Acrivastine	DB09488	Escherichia Coli Septicemia	C0276088
Acrivastine	DB09488	Miscarriage With Sepsis	C0269398
Acrivastine	DB09488	Erectile Dysfunction	C0242350
Sodium ferric gluconate complex	DB09517	Chlamydial Urethritis	C1278807
Sodium ferric gluconate complex	DB09517	Acute Bacterial Sinusitis	C0275556
Sodium phosphate, monobasic, monohydrate	DB09525	Rhinitis	C0035455
Sodium phosphate, monobasic, monohydrate	DB09525	Acute Gonococcal Cervicitis	C0153195
Dexchlorpheniramine maleate	DB09555	Gonorrhea of Pharynx	C0149966

Continued on next page

Table 2 – continued from previous page

Drug name	DrugBank ID	Disease name	Disease CUI
Bilastine	DB11591	Tularemia	C0041351
Bilastine	DB11591	Glaucoma, Open-Angle	C0017612
Bilastine	DB11591	Chronic Tubotympanic Suppurative Otitis Media	C0155440
Bilastine	DB11591	Septicemia Candida	C0349009
Bilastine	DB11591	Arthritis, Gouty	C0003868
Bilastine	DB11591	Complex Dyslipidemia	C3875286
Levoleucovorin	DB11596	Irritable Bowel Syndrome Characterized By Constipation	C1868889

Table 2: SNF-NN predicted drug-disease interactions

References

[1] D. S. Wishart, Y. D. Feunang, A. C. Guo, E. J. Lo, A. Marcu, J. R. Grant, T. Sajed, D. Johnson, C. Li, Z. Sayeeda *et al.*, "Drugbank 5.0: a Major Update to the Drugbank Database for 2018," *Nucleic Acids Research*, vol. 46, no. D1, pp. D1074–D1082, 2017.

[2] Y. Wang, S. Zhang, F. Li, Y. Zhou, Y. Zhang, Z. Wang, R. Zhang, J. Zhu, Y. Ren, Y. Tan *et al.*, "Therapeutic Target Database 2020: Enriched Resource for Facilitating Research and Early Development of Targeted Therapeutics," *Nucleic Acids Research*, p. 1, 2019.

[3] T. Hernandez-Boussard, M. Whirl-Carrillo, J. M. Hebert, L. Gong, R. Owen, M. Gong, W. Gor, F. Liu, C. Truong, R. Whaley *et al.*, "The Pharmacogenetics and Pharmacogenomics Knowledge Base: Accentuating the Knowledge," *Nucleic Acids Research*, vol. 36, no. suppl_1, pp. D913–D918, 2007.

[4] A. Gaulton, A. Hersey, M. Nowotka, A. P. Bento, J. Chambers, D. Mendez, P. Mutowo, F. Atkinson, L. J. Bellis, E. Cibrián-Uhalte *et al.*, "The ChEMBL Database in 2017," *Nucleic Acids Research*, vol. 45, no. D1, pp. D945–D954, 2016.

[5] A. J. Pawson, J. L. Sharman, H. E. Benson, E. Faccenda, S. P. Alexander, O. P. Buneman, A. P. Davenport, J. C. McGrath, J. A. Peters, C. Southan *et al.*, "The IUPHAR/BPS Guide to PHARMACOLOGY: an Expert-driven Knowledgebase of Drug Targets and Their Ligands," *Nucleic Acids Research*, vol. 42, no. D1, pp. D1098–D1106, 2014.

[6] T. Liu, Y. Lin, X. Wen, R. N. Jorissen, and M. K. Gilson, "BindingDB: a Web-accessible Database of Experimentally Determined Protein–ligand Binding Affinities," *Nucleic Acids Research*, vol. 35, no. suppl_1, pp. D198–D201, 2007.

[7] T. U. Consortium, "UniProt: The Universal Protein Knowledgebase," *Nucleic Acids Research*, vol. 45, no. D1, pp. D158–D169, 2016.

[8] L. Breuza, S. Poux, A. Estreicher, M. L. Famiglietti, M. Magrane, M. Tognolli, A. Bridge, D. Baratin, and N. Redaschi, "The UniProtKB Guide to the Human Proteome," *Database*, vol. 2016, 2016.

[9] M. Kuhn, I. Letunic, L. J. Jensen, and P. Bork, "The SIDER Database of Drugs and Side Effects," *Nucleic Acids Research*, vol. 44, no. D1, pp. D1075–D1079, 2015.

[10] F. Cheng, W. Li, Z. Wu, X. Wang, C. Zhang, J. Li, G. Liu, and Y. Tang, "Prediction of Polypharmacological Profiles of Drugs by the Integration of Chemical, Side effect, and Therapeutic Space," *Journal of Chemical Information and Modeling*, vol. 53, no. 4, pp. 753–762, 2013.

[11] M. Kuhn, M. Campillos, I. Letunic, L. J. Jensen, and P. Bork, "A Side Effect Resource to Capture Phenotypic Effects of Drugs," *Molecular Systems Biology*, vol. 6, no. 1, 2010.

[12] A. P. Davis, B. L. King, S. Mockus, C. G. Murphy, C. Saraceni-Richards, M. Rosenstein, T. Wiegers, and C. J. Mattingly, "The Comparative Toxicogenomics Database: Update 2011," *Nucleic Acids Research*, vol. 39, no. suppl_1, pp. D1067–D1072, 2010.

[13] C. E. Lipscomb, "Medical Subject Headings (MeSH)," *Bulletin of the Medical Library Association*, vol. 88, no. 3, p. 265, 2000.

[14] O. Bodenreider, "The Unified Medical Language System (UMLS): Integrating Biomedical Terminology," *Nucleic Acids Research*, vol. 32, no. suppl_1, pp. D267–D270, 2004.

[15] S. Kim, P. A. Thiessen, E. E. Bolton, J. Chen, G. Fu, A. Gindulyte, L. Han, J. He, S. He, B. A. Shoemaker *et al.*, "Pubchem Substance and Compound Databases," *Nucleic Acids Research*, vol. 44, no. D1, pp. D1202–D1213, 2015.

[16] C. Steinbeck, Y. Han, S. Kuhn, O. Horlacher, E. Luttmann, and E. Willighagen, "The Chemistry Development Kit (CDK): an open–source Java Library for Chemo– and Bioinformatics," *Journal of Chemical Information and Computer Sciences*, vol. 43, no. 2, pp. 493–500, 2003.

[17] G. O. Consortium, "The Gene Ontology Resource: 20 Years and Still Going Strong," *Nucleic Acids Research*, vol. 47, no. D1, pp. D330–D338, 2019.

[18] P. Resnik, "Semantic Similarity in a Taxonomy: an Information-based Measure and its Application to Problems of Ambiguity in Natural Language," *Journal of Artificial Intelligence Research*, vol. 11, pp. 95–130, 1999.

[19] D. S. Wishart, Y. D. Feunang, A. Marcu, A. C. Guo, K. Liang, R. Vázquez-Fresno, T. Sajed, D. Johnson, C. Li, N. Karu *et al.*, "HMDB 4.0: The Human Metabolome Database for 2018," *Nucleic Acids Research*, vol. 46, no. D1, pp. D608–D617, 2018.

[20] E. Boutet, D. Lieberherr, M. Tognolli, M. Schneider, P. Bansal, A. J. Bridge, S. Poux, L. Bougueleret, and I. Xenarios, "UniProtKB/Swiss-Prot, the Manually Annotated Section of the UniProt KnowledgeBase: How to Use the Entry View," in *Plant Bioinformatics*. Springer, 2016, pp. 23–54.

[21] M. Kanehisa and S. Goto, "KEGG: Kyoto Encyclopedia of Genes and Genomes," *Nucleic Acids Research*, vol. 28, no. 1, pp. 27–30, 2000.

[22] T. F. Smith, M. S. Waterman, and C. Burks, "The Statistical Distribution of Nucleic Acid Similarities," *Nucleic Acids Research*, vol. 13, no. 2, pp. 645–656, 1985.

[23] A. P. Davis, C. J. Grondin, R. J. Johnson, D. Sciaky, R. McMorran, J. Wiegers, T. C. Wiegers, and C. J. Mattingly, "The Comparative Toxicogenomics Database: Update 2019," *Nucleic Acids Research*, vol. 47, no. D1, pp. D948–D954, 2019.

[24] H. L. Rehm, J. S. Berg, L. D. Brooks, C. D. Bustamante, J. P. Evans, M. J. Landrum, D. H. Ledbetter, D. R. Maglott, C. L. Martin, R. L. Nussbaum *et al.*, "ClinGen—-the Clinical Genome Resource," *New England Journal of Medicine*, vol. 372, no. 23, pp. 2235–2242, 2015.

[25] S. Köhler, L. Carmody, N. Vasilevsky, J. O. B. Jacobsen, D. Danis, J.-P. Gourdine, M. Gargano, N. L. Harris, N. Matentzoglu, J. A. McMurry *et al.*, "Expansion of the Human Phenotype Ontology (HPO) Knowledge Base and Resources," *Nucleic Acids Research*, vol. 47, no. D1, pp. D1018–D1027, 2019.

[26] IBI Group. (2020, July) BeFree. [Online]. Available: http://ibi.imim.es/tools/befree/

[27] A. Bravo, M. Cases, N. Queralt-Rosinach, F. Sanz, and L. Furlong, "A Knowledge-driven Approach to Extract Disease-related Biomarkers from the Literature," *BioMed Research International*, vol. 2014, 2014.

[28] À. Bravo, J. Piñero, N. Queralt-Rosinach, M. Rautschka, and L. I. Furlong, "Extraction of Relations Between Genes and Diseases from Text and Large-scale Data Analysis: Implications for Translational Research," *BMC Bioinformatics*, vol. 16, no. 1, p. 55, 2015.

[29] M. J. Li, Z. Liu, P. Wang, M. P. Wong, M. R. Nelson, J.-P. A. Kocher, M. Yeager, P. C. Sham, S. J. Chanock, Z. Xia *et al.*, "GWASdb v2: an Update Database for Human Genetic Variants Identified by Genome-wide Association Studies," *Nucleic Acids Research*, vol. 44, no. D1, pp. D869–D876, 2016.

[30] S. T. Sherry, M.-H. Ward, M. Kholodov, J. Baker, L. Phan, E. M. Smigielski, and K. Sirotkin, "dbSNP: The NCBI Database of Genetic Variation," *Nucleic Acids Research*, vol. 29, no. 1, pp. 308–311, 2001.

[31] P. Thomas, T. Rocktäschel, J. Hakenberg, Y. Lichtblau, and U. Leser, "SETH Detects and Normalizes Genetic Variants in Text," *Bioinformatics*, vol. 32, no. 18, pp. 2883–2885, 2016.

[32] R. E. Drury, D. O'Connor, and A. J. Pollard, "The Clinical Application of microRNAs in Infectious Disease," *Frontiers in Immunology*, vol. 8, p. 1182, 2017.

[33] L. Cheng, Y. Hu, J. Sun, M. Zhou, and Q. Jiang, "DincRNA: a Comprehensive Web-based Bioinformatics Toolkit for Exploring Disease Associations and ncRNA Function," *Bioinformatics*, vol. 34, no. 11, pp. 1953–1956, 2018.

[34] R.-W. Yao, Y. Wang, and L.-L. Chen, "Cellular Functions of Long Noncoding RNAs," *Nature Cell Biology*, vol. 21, no. 5, pp. 542–551, 2019.

[35] N. Freimer and C. Sabatti, "The Human Phenome Project," *Nature Genetics*, vol. 34, no. 1, pp. 15–21, 2003.

[36] X. Wu, Q. Liu, and R. Jiang, "Align human interactome with phenome to identify causative genes and networks underlying disease families," *Bioinformatics*, vol. 25, no. 1, pp. 98–104, 2009.

[37] A. Gottlieb, G. Y. Stein, E. Ruppin, and R. Sharan, "PREDICT: a Method for Inferring Novel Drug Indications with Application to Personalized Medicine," *Molecular Systems Biology*, vol. 7, no. 1, 2011.

[38] G. K. Mazandu, E. R. Chimusa, and N. J. Mulder, "Gene Ontology Semantic Similarity Tools: Survey on Features and Challenges for Biological Knowledge Discovery," *Briefings in Bioinformatics*, vol. 18, no. 5, pp. 886–901, 2017.

[39] "Network-based elucidation of human disease similarities reveals common functional modules enriched for pluripotent drug targets."

[40] J. Piñero, J. M. Ramírez-Anguita, J. Saüch-Pitarch, F. Ronzano, E. Centeno, F. Sanz, and L. I. Furlong, "The disgenet knowledge platform for disease genomics: 2019 update," *Nucleic acids research*, vol. 48, no. D1, pp. D845–D855, 2020.

[41] P. Resnik, "Using Information Content to Evaluate Semantic Similarity in A Taxonomy," *arXiv preprint cmp-lg/9511007*, 1995.

[42] D. Lin *et al.*, "An Information-theoretic Definition of Similarity," in *ICML*, vol. 98, no. 1998, 1998, pp. 296–304.

[43] J. Z. Wang, Z. Du, R. Payattakool, P. S. Yu, and C.-F. Chen, "A New Method to Measure the Semantic Similarity of GO Terms," *Bioinformatics*, vol. 23, no. 10, pp. 1274–1281, 2007.

[44] S. Mathur and D. Dinakarpandian, "Finding Disease Similarity Based on Implicit Semantic Similarity," *Journal of Biomedical Informatics*, vol. 45, no. 2, pp. 363–371, 2012.

[45] L. Cheng, J. Li, P. Ju, J. Peng, and Y. Wang, "SemFunSim: a New Method for Measuring Disease Similarity by Integrating Semantic and Gene Functional Association," *PLOS One*, vol. 9, no. 6, p. e99415, 2014.
